# CryoEM Structures of Native Quinol-Dependent Nitric Oxide Reductase in Resting and Quinol-Bound States

**DOI:** 10.1021/acsbiomedchemau.5c00245

**Published:** 2026-03-13

**Authors:** Faisal T. Khaja, Allegra Mboukou, Louie P. Aspinall, Charlotte E. Hawksworth, Robert R. Eady, Svetlana V. Antonyuk, Stephen P. Muench, S. Samar Hasnain

**Affiliations:** † Department of Biochemistry, Cell and Systems Biology, Institute of Systems, Molecular and Integrative Biology, 4591 University of Liverpool, Liverpool L69 7ZB, U.K.; ‡ School of Molecular and Cellular Biology, Faculty of Biological Sciences, 4468 University of Leeds, Leeds LS2 9JT, U.K.; § Astbury Centre for Structural Molecular Biology, University of Leeds, Leeds LS2 9JT, U.K.; ∥ School of Biomedical Sciences, Faculty of Biological Sciences, University of Leeds, Leeds LS2 9JT, U.K.

**Keywords:** denitrification, proton transfer, cryo-electron microscopy, electron transfer, quinol binding, nitrous oxide, alphaFold3

## Abstract

The membrane-bound quinol-dependent nitric oxide reductases (qNORs), which are members of the respiratory heme-copper oxidase superfamily, are of major importance to food production, environment, and human health. They are unique to bacteria and catalyze N–N bond formation, converting nitric oxide (NO) to generate the enzymatic product, nitrous oxide (N_2_O), in agricultural and pathogenic conditions. High-resolution qNOR structures have been reported from two bacterial species, in which the molecular size of the protein was increased by the insertion of apocytochrome b_562_ (BRIL) at the C-terminus to facilitate cryoEM structure determination. However, it remains uncertain how BRIL fusion alters the native structure of these metalloenzymes. Here, we present the first high-resolution structure of *Achromobacter xylosoxidans* qNOR (*Ax*qNOR) determined without a fusion tag at two different pH values, revealing structural differences near the catalytic core as well as overall conformational changes between the native and fusion-tagged structures. The native enzyme shows a bell-shaped pH dependence of enzymatic activity, like nitrite reductase, the preceding enzyme in the denitrification pathway, which generates the substrate NO. In addition, we report structures of *Ax*qNOR bound to quinol and hydroxyquinol that provide valuable insight into the potential electron transfer pathway originating from Trp718 to the redox centers.

## Graphical abstract

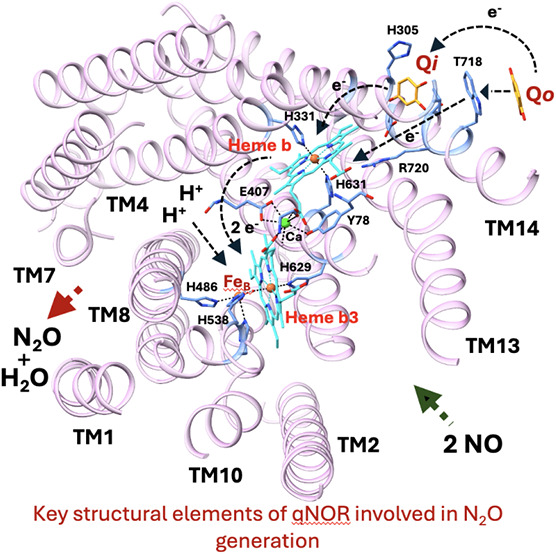

## Introduction

Nitric oxide reductases (NORs) are enzymes that catalyze the reaction 2 NO + 2H^+^ + 2*e* ^–^ → N_2_O + H_2_O and play a central role in denitrification, a microbial energy-yielding pathway where NO_3_^–^ is reduced to N_2_ in a series of consecutive redox reactions (NO_3_^–^ → NO_2_^–^ → NO → N_2_O → N_2_) mediated by different metalloenzymes.^1–5^ The reaction occurs at a binuclear active site composed of a high-spin heme *b*_*3*_and a nonheme iron (Fe_B_), which together bind and couple two NO molecules to form the N–N bond.^6^ Electrons required for catalysis are transferred to the active site *via* low-spin heme *b,* and a conserved Ca^2+^ ion stabilizes the structural interface and contributes to maintaining the structural integrity of the catalytic center.^1–5^ The denitrification pathway is part of the global nitrogen cycle, with agronomic (losses of soil nitrogen available for crop growth-the latest estimate is up to 50% loss of applied N fertilizer) and environmental (generation of the potent ozone-depleting and greenhouse gas N_2_O) consequences.^7,8^ Two major types of membrane-bound bacterial NORs have been characterized: the cytochrome *bc*-type (cNOR), a heterodimeric enzyme encoded by *NorBC* that receives electrons from soluble redox protein donors, and the quinol-dependent (qNOR), a single-subunit enzyme encoded by *NorZ* that lacks the cytochrome *c* subunit and uses ubiquinol as the electron donor.^2,9^ qNORs also have medical relevance, as they are present in many nondenitrifying pathogenic microorganisms, including *Corynebacterium diphtheriae*, *Neisseria gonorrheae*, *Neisseria meningitidis*, and *Staphylococcus aureus*, where they help detoxify NO produced by the host’s defense system, enabling these pathogens to evade the host’s immune response.^7,10–13^ For example, the pathogen *N. meningitidis* shows depleted survival in nasopharyngeal tissue when *Nm*qNOR is knocked out.^14^ The formation of the N–N bond in nature poses chemical challenges due to the high electronegativity of nitrogen. NORs achieve this efficiently, but how this is achieved remains largely unanswered due to limited structural information that can be related to functional data. Over the past decade, several crystallographic structures of NORs, including qNORs, have been reported. These structures revealed the organization and structures of the redox centers, heme *c* in NorC, and heme *b* in NorB and NorZ. They also revealed the retention of the structure of the binuclear catalytic center-heme *b*_3_ and nonheme iron Fe_B_ in both cNOR and qNORs.^15–20^ Size-exclusion chromatography of purified *Nm*qNOR revealed a dynamic equilibrium between monomeric and dimeric states. While the monomeric fraction could be crystallized and its structure determined by X-ray crystallography, the dimeric species failed to yield crystals. Similarly, *Achromobacter xylosoxidans* qNOR (*Ax*qNOR) displayed pronounced polydispersity, with monomeric and dimeric forms in dynamic equilibrium, rendering it unsuitable for X-ray crystallography.^15,21^ All of these crystallographic structures showed a monomeric assembly, some of which have been shown to be artifacts resulting from the use of divalent atoms, e.g., Zn, during crystallization.^15,16^

Over the last five years, cryoEM structures of qNORs from *Alcaligenes xylosoxidans* (*Ax*qNOR) and *Neisseria meningitidis* (*Nm*qNOR) have been reported, providing the first information on the dimeric assemblies of NORs and how they might be destabilized.^21–24^ The highest-resolution cryoEM structure of *Ax*qNOR is ∼2.2 Å, a resolution considered sufficient to address the processes that underpin catalysis in metalloenzymes.^23^ For these cryoEM structures, apocytochrome *b*_562_ (BRIL_562_) was used as a fusion partner in order to increase the molecular weight, as the monomer was expected to be ∼85 kDa. The fusion was achieved by truncating the C-terminal of qNORs by 16 residues. Fusion with BRIL has been particularly successful in the crystallography of GPCRs, along with T4 lysozyme.^25,26^ In addition to BRIL, fusion with a thermostable glycogen synthase domain from *Pyrococcus abyssi* (PGS) (MW ∼20 kDa) has been explored for cryoEM studies of GPCRs.^25^ However, it remains uncertain how such fusion alters the native structure of proteins. Despite the shortcomings mentioned above, these structures have provided important insights into the overall architecture of these important enzymes but also have established the nature of the catalytic core. The structure of BRIL-*Ax*qNOR consists of 18 α-helices packed together into each monomer, with the dimer interface created by transmembrane helices 2 and 11. Overall, the monomer arrangement of *Ax*qNOR is very similar to the arrangement of NorB, the catalytic domain of cNOR, with the main features of the catalytic core and ET heme preserved.^18^ The heme *b* (electron transfer heme) and the binuclear Fe_**B**_-heme *b_3_* centers (catalytic core) are linked by a calcium ion, which may help to correctly position these centers for efficient ET during a catalytic cycle.^23^ However, it remains uncertain how BRIL fusion alters the native structure of these metalloenzymes.

We note that since the original cryoEM structure determination of qNORs, several advances in experimental and single-particle image processing have pushed the lower molecular weight boundary significantly, as such making use of fusion proteins unnecessary. Despite this progress, structure determination by cryoEM remains challenging for smaller proteins (<50 kDa), which account for less than 2% of all reconstructions deposited in the Electron Microscopy Data Bank (EMDB). However, structure determination for 50–100 kDa proteins (currently ∼6.5%) and 100–250 kDa (∼32%) is steadily increasing (www.ebi.ac.uk/emdb/).

Here, we present high-resolution cryoEM structures of the native full-length *Ax*qNOR without any BRIL fusion and reevaluated the previously published cryoEM structures of both *Ax*qNOR and *Nm*qNOR to tease out the interaction between BRIL and qNORs and how this may affect the overall conformation of the enzyme.

Enzymatic activity, including that for NORs, is affected by pH and results from changes in the protonation states of the amino acid residues both in the catalytic pocket and those involved in proton delivery. The pH dependence for *Pseudomonas nautica* cNOR (*Pn*cNOR) *and Nm*qNOR has been shown to have bell-shaped curves.^15,27^ In the case of *Pn*cNOR the enzyme was immobilized on a rotating graphite disk electrode, for which a broad pH optimum of 4–7.5 was observed, while for *Nm*qNOR, a pH optimum of ∼7 was observed; but in this case, a variety of buffers were used to cover the pH range.^15,27^ In the case of cNOR, it has been suggested that five protonable groups in the vicinity of the catalytic binuclear center modulate enzyme activity as a function of pH.^27^

In this study, we determined cryoEM structures of native *Ax*qNOR at pH 6.5 and pH 8.0 and show that its enzymatic activity exhibits a distinct bell-shaped pH dependence, with maximum activity at pH 6.5. The pH-dependence curve is very similar to copper nitrite reductases, where two proton-donating residues have been assigned.^28^ We show that fusion with BRIL causes significant conformational changes, while the change of pH in the native enzyme only marginally impacts the catalytic core and its surroundings. In addition, we determined cryoEM structures of *Ax*qNOR-bound quinol and hydroxyquinol, revealing putative ubiquinol-binding sites and a possible electron transfer pathway from Trp718 to the heme *b* redox center.

### Structure Determination of Native *Ax*qNOR

The cryoEM structure of the native *Ax*qNOR was determined at pH 8 and pH 6.5 using freshly purified samples, resulting in a reconstruction in the range of 2.3–2.6 Å resolution. Each *Ax*qNOR protomer contains 14 transmembrane helices (TM), with residues from TM2 (residues 230–252), TM10 (residues 546–568), TM11 (residues 589–611), and TM13 (residues 666–688) from Chain A and Chain B contributing to the dimeric interface. The redox centers in *Ax*qNOR are clearly resolved in the cryoEM density map, which are located within the core of the protein between TM3 (residues 277–309), TM4 (residues 324–355), TM8 (residues 473–508), TM9 (residues 513–538), TM11 (residues 581–611), and TM12 (residues 624–653) (Figure 1). These include the electron-accepting heme *b* and the binuclear active site composed of heme *b*_*3*_ and a nonheme iron ion (Fe_B_), which are connected *via* a μ-oxo bridge. In addition, a calcium ion that bridges heme *b* and heme *b*_*3*_ is also well-defined, indicating a structurally stabilized architecture of the redox centers (Figure 1). The spatial arrangement of these redox centers, together with key catalytic residues, establishes a redox potential gradient that finely modulates electron transfer from ubiquinol in the membrane to heme *b*, and subsequently to the binuclear high-spin heme *b_3_*-Fe_B_ catalytic unit, where the reduction of nitric oxide (NO) to nitrous oxide (N_2_O) takes place. The EM density for most of the transmembrane region was sufficiently well resolved to enable confident model building, except for TM1 (residues 4–30) and TM7 (residues 445–461), which were only discernible at very low-density thresholds (Supplemental Figure S1). Model building of these helices was assisted by visual inspection of sharpened and blurred maps generated by converting map’s MRC format to MTZ in CCP-EM with different B-factors, as well as by maps obtained from deep learning-based approaches such as DeepEMhancer.^29,30^

**Figure 1:**
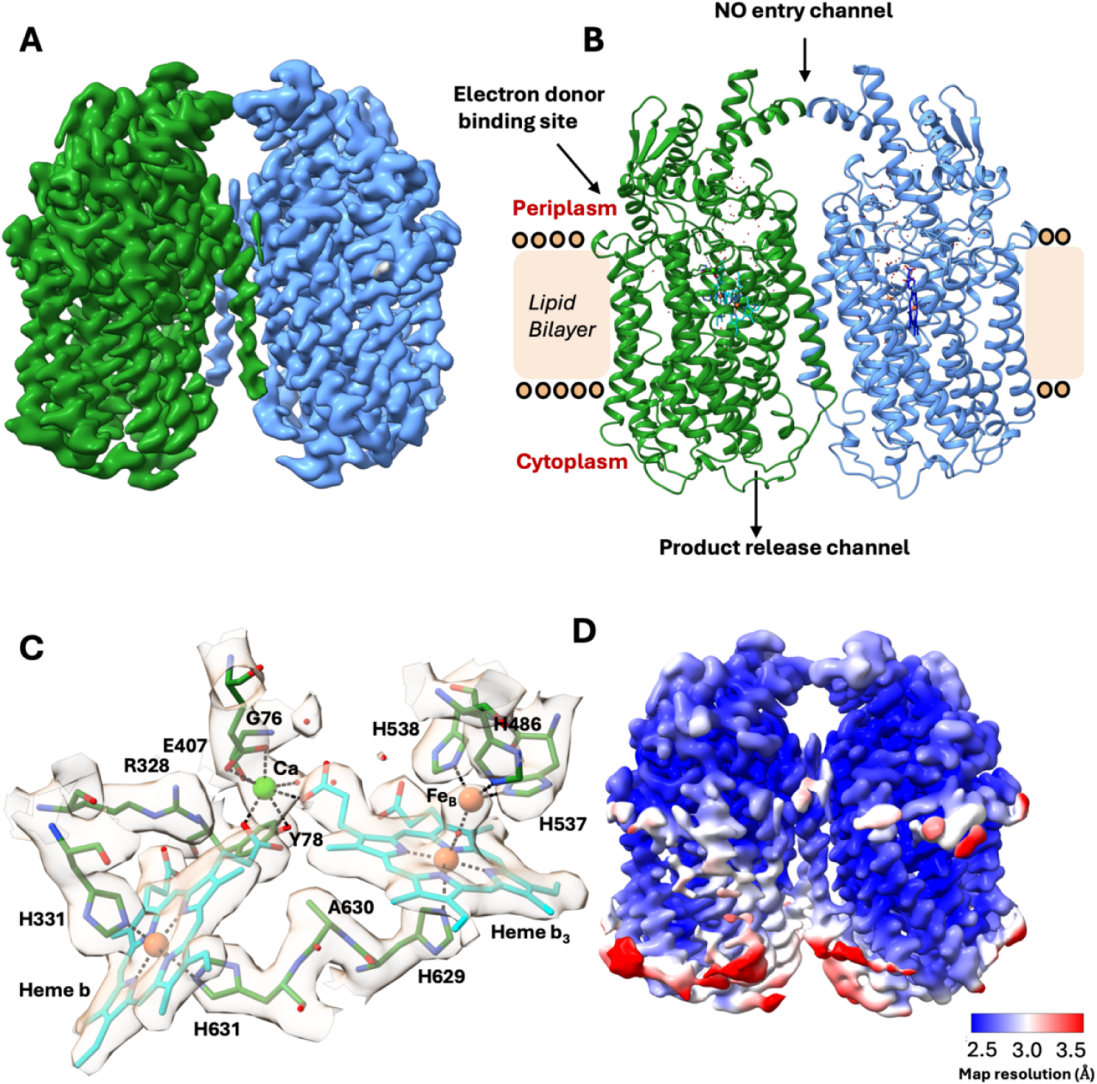
CryoEM structure of native *Ax*qNOR. (A) CryoEM Coulomb potential map of the native AxqNOR determined by single-particle cryoEM at pH 8.0 to an overall resolution of 2.7 Å. The map is colored by protomer (chain A, green, and chain B, blue). (B) Dimeric *Ax*qNOR oriented in the plane of the lipid bilayer. Water molecules are shown as red dots. The putative electron donor site, NO entry channel (on the periplasmic side), and product release channel (on the cytoplasmic side) are indicated. (C) Binuclear center with corresponding cryoEM density showing heme *b* and heme *b*_3_ (cyan sticks), the Ca^2+^ ion (green sphere), and the nonheme Fe^_B_^ atom (coral). Ca^2+^ interacts with heme propionates, Glu407, Gly76, and Tyr78. Fe^_B_^ is coordinated by His486, His537, and His538 and forms a μ-oxo bridge with heme *b*_3_. Heme *b* is coordinated by His331 and His631. (D) CryoEM density map colored by local resolution.

In *Ax*qNOR, heme *b* is coordinated by His331 and His631, forming a six-coordinate low-spin iron center, while the imidazole ring of His629 serves as the axial ligand for high-spin heme *b_3_*. The nonheme metal ion (Fe_B_) in *Ax*qNOR is coordinated by His538, His537, and His486, and Ca^2+^, which links heme *b* and heme *b_3_* ensures their precise spatial orientation throughout the catalytic cycle. In *Ax*qNOR, the Ca^2+^ ion is coordinated by seven oxygen atoms in a well-defined octahedral geometry.

These include the backbone oxygen of Gly76, the hydroxyl group of Tyr78, the carboxylate oxygens (OE1 and OE2) of Glu407, the O1D and O2A propionates of heme *b* and heme *b_3_,* and a water molecule, with bond lengths ranging from 2.2 to 2.8 Å (Figure 1). During cryoEM data collection of native *Ax*qNOR, we consistently observed an additional density between the periplasmic residues His217 and His224 when samples were vitrified on Quantifoil copper (Cu) grids, which was modeled as a bound copper ion (Supplemental Figure S1). This density was absent when identical preparations were imaged on gold (Au) grids. Copper supports can release trace Cu^2+^ ions during glow discharge and sample application, and the imidazole side chains of His217 and His224 provide a favorable site for chelating Cu ions. In contrast, elemental gold is chemically inert under such conditions and does not generate soluble Au species, explaining the lack of a corresponding gold atom or coordinating density in data sets collected with Au grids. These findings highlight how grid composition can introduce adventitious metal binding particularly when high affinity metal binding ligands may be available and thus should be considered when interpreting cryoEM structures.

*Ax*qNOR contains five catalytically important glutamate residues: Glu407, Glu410, Glu490, Glu494, and Glu559. Three of these residues—Glu490, Glu494, and Glu559—are near the nonheme Fe_B_ of the binuclear catalytic center, while Glu407 coordinates the Ca^2+^ ion, and Glu410 interacts with a key water molecule located near heme *b*.^23^ All five Glu and His residues ligating the heme are functionally important for the catalytic NO reduction and are conserved among homologous qNOR and cNOR enzymes (Supplemental Figure S2).^31^ The spatial arrangement of the Glu side chains and their coordination sphere with waters and the nonheme metal ion exhibits a high degree of structural variability in side-chain conformation, likely altering the catalytic state of the enzyme.^15–20^ This flexible coordination is critical for NO reduction in NORs, as it facilitates substrate accommodation and mediates proton transfer required for N–O bond cleavage during the conversion of nitric oxide (NO) to nitrous oxide (N_2_O).^31^

Structural superposition of *Ax*qNOR with homologous NORs was performed using the heme *b* coordinates as the least-squares (LSQ) reference, effectively “locking” the alignment to the redox-center framework and enabling direct visualization of local differences near the catalytic binuclear site (heme *b_3_*-nonheme metal). In parallel, a chain-based superposition was carried out to assess global structural differences, revealing that the overall transmembrane topology and redox-center arrangement of native *Ax*qNOR closely resemble those of homologous nitric oxide reductases: *Neisseria meningitidis* (*Nm*qNOR; PDB 6L1X; rmsd ∼1.5 Å for 740 Cα atoms), *Geobacillus stearothermophilus* (*Gs*qNOR; PDB 3AYG; ∼2.2 Å for 719 Cα atoms), *Pseudomonas aeruginosa* cNOR (*Pac*NOR; PDB 3O0R; ∼2.3 Å for 430 Cα atoms), and *Roseobacter dentrificans* (*Rdc*NOR; PDB 4XYD; ∼2.2 Å for 433 Cα atoms). Notable differences, however, occur near the heme *b* *_3_-*nonheme metal binuclear center. For example, the distance between heme *b_3_* and the nonheme metal, where the NO reduction takes place, varies among homologous NORs-measuring ∼3.3 Å in *Ax*qNOR, 4.5 Å in *Gs*qNOR, 3.9 Å in *Nm*qNOR, 4.3 Å in *Rd*cNOR, and 3.8 Å in *Pa*cNOR. While Fe_B_ serves as the nonheme metal ion in *Ax*qNOR, *Nm*qNOR, *Pa*cNOR, and *Rd*cNOR, it is replaced by Zn in *Gs*qNOR, likely introduced by the crystallization reagent and results in a loss of activity. Notably, the nonheme metal ion adopts a tetrahedral geometry in *Ax*qNOR, *Nm*qNOR and *Gs*qNOR, octahedral geometry in *Rd*cNOR and a slightly distorted trigonal-bipyramidal geometry in *Pa*cNOR (Supplementary Figure S2). These local variations in active-site architecture may underlie the observed differences in NO-reduction rates across NORs, potentially conferring adaptive advantages to their respective host organisms (Supplemental Figure S3). Alternatively, they may indicate subtle differences in the redox states of different enzymes’ samples.

Structural superposition of BRIL-*Ax*qNOR (previously reported *Ax*qNOR structure with BRIL fusion, PDB: 8BGW) and the native *Ax*qNOR revealed a root-mean-square deviation (rmsd) of ∼2.0 Å across Cα 750 positions, indicating notable conformational differences. In contrast, BRIL-*Ax*qNOR showed higher similarity to BRIL-*Nm*qNOR (PDB: 8ZGP; rmsd ∼1.2 Å across Cα 739 positions), suggesting that BRIL fusion induces comparable conformational changes in qNOR that substantially reshape the overall structure of the native qNOR (Figure 2). While the BRIL fusion has so far provided unprecedented structural detail of qNOR enzymes, it is now clear that BRIL-mediated interactions introduce structural artifacts that could bias structural and functional characterization. This consideration is particularly important when interpreting the functional consequences of the observed architecture, especially in the context of engineered point mutations aimed at probing structural rearrangements and elucidating the mechanistic basis of qNOR activity.

**Figure 2:**
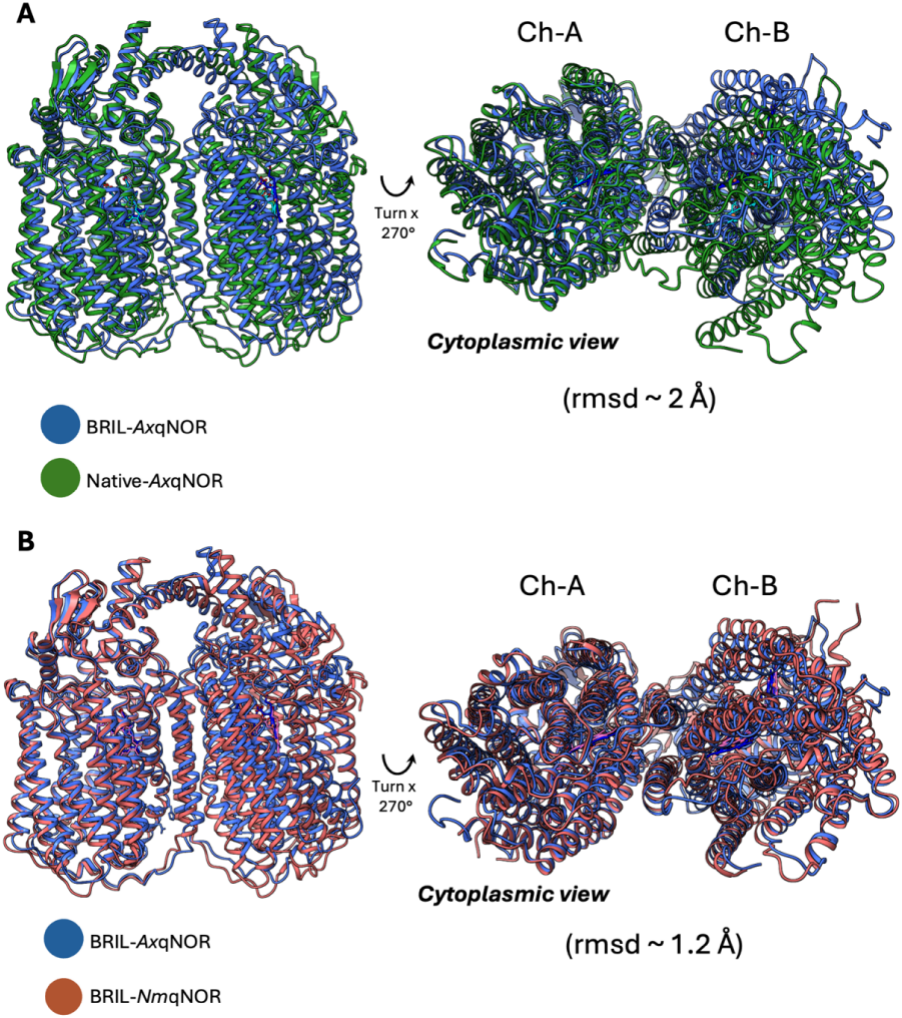
Structural superposition of native and BRIL-*Ax*qNOR. (A) Superposition of BRIL-*Ax*qNOR with native *Ax*qNOR shows an rmsd of ∼2.0 Å across Cα 750 residues, indicating notable conformational differences. (B) BRIL-*Ax*qNOR aligns more closely with BRIL-*Nm*qNOR (PDB 8ZGP; rmsd of ∼1.2 Å across Cα 739 residues), suggesting that BRIL fusion induces similar structural rearrangements and may introduce artifacts that affect functional interpretation.

To understand the basis of this conformational change, we reanalyzed our BRIL-*Ax*qNOR cryoEM data sets and used AlphaFold-predicted BRIL fusion models to guide and assess BRIL conformation. Finally, a detailed structural comparison with native *Ax*qNOR was performed to identify the differences.

### CryoEM Structure Determination of BRIL-*Ax*qNOR and Comparison with Native *Ax*qNOR

The BRIL-*Ax*qNOR structure published by Gopalasingam et al. showed only faint BRIL density.^22^ However, reprocessing the same micrographs with an updated cryoEM workflow produced a 3.4 Å 3D reconstruction from ∼85,000 particles, which is an improvement over the original 3.9 Å map obtained from ∼44,000 particles, also revealing a better-defined BRIL density (Supplemental Figure S4). Interestingly, a 2.2 Å data set collected from the same batch of grids that were stored for three years showed no BRIL density, even though the overall qNOR structure largely remained unchanged.^23^ We suggest the loss of BRIL signal likely arises from a combination of specimen preparation and experimental factors. Small peripheral and flexible domains, such as BRIL, are particularly vulnerable to limited proteolysis during long-term cryogenic storage and mechanical damage at the air–water interface. These processes increase conformational heterogeneity and reduce the fraction of particles presenting BRIL in a consistent orientation, thereby weakening its averaged signal. Additionally, subtle changes in local ice thickness or foil integrity over time can alter beam-induced motion and particle orientation distributions, disproportionately affecting low-mass, flexible domains like BRIL while leaving the rigid qNOR core largely unaffected. Beyond specimen-related changes, variations in data collection parameters may cause radiolytic damage, significantly influencing the detectability of weak or heterogeneous densities.

As a consequence, a feature resolved under one set of conditions may become undetectable under another. For example, Jamali *et al.* observed a faint BRIL density in the structure of BRIL-*Nm*qNOR resolved at 3.15 Å (PDB 6L3H; EMD-0822), whereas Gopalasingam et al. detected no BRIL density in a structure resolved at 1.9 Å (PDB 8ZGP; EMD-60086), using the same BRIL-*Nm*qNOR construct with a completely different experimental setup.^21,24^ Together, these cases point to BRIL’s heightened sensitivity to subtle changes in vitrified specimens and experimental conditions. A systematic study, which is beyond the scope of the current study, is required to establish the key factors.

Apart from the BRIL, the overall structure of the reprocessed BRIL-*Ax*qNOR at 3.4 Å is consistent with the original structures.^22,23^ However, due to the diffuse, low-resolution nature of the BRIL density, its *de novo* model building was not feasible. Instead, an AlphaFold-predicted BRIL model was rigid-body fitted into the cryoEM map to infer its orientation. While this approach does not resolve fine structural details, it provides a reliable estimate of BRIL’s spatial position and possible interactions with qNOR. Among five AlphaFold3-predicted BRIL–*Ax*qNOR models, one exhibited the highest model-to-map correlation coefficient (∼0.7) when rigid-body fitted into the cryoEM map (contoured at 0.15 σ) (Supplemental Figures S4 and S5). This model offered the best overall fit at this resolution, enabling the better placement of the BRIL domain within the EM density compared with the other predictions.

The model predicted multiple ionic interactions between BRIL and the C-terminal region of *Ax*qNOR, including Lys364-Asp758, Lys364-Glu754, Arg280-Glu754, Arg280-Glu750, Asp751-Arg654, Gln656-Asp748, Arg267-Glu795, and Asp785-Asn752. These contacts occur at the BRIL-qNOR interface and likely contribute to structural stabilization (Figure 3A). Structural comparisons between native *Ax*qNOR and BRIL-*Ax*qNOR indicate that the monomeric fold is largely preserved, showing only minor deviations in secondary structure and core domain arrangement. In contrast, the dimeric assembly exhibits pronounced conformational differences, suggesting that the BRIL fusion may influence intersubunit packing or quaternary dynamics, thereby altering the overall protein architecture. For example, the total buried area of native *Ax*qNOR is 15,917 Å^2^, whereas in BRIL-*Ax*qNOR it increases to 23,031 Å^2^, representing a 44% increase. Similarly, the buried surface area between Chain A and Chain B rises from 1,795 Å^2^ in native *Ax*qNOR to 2,011 Å^2^ in BRIL-*Ax*qNOR, approximately 12% increase, as calculated by the PISA server.^32^ These changes are manifested in the rearrangement of helices, which affects interactions among TM2, TM3, TM4, and PM4 (residues 113–128 in the periplasmic region)-key elements for maintaining dimer integrity (Supplementary Figure S6). As a result, the quaternary structures exhibit significant divergence in their dimeric architecture between BRIL and native *Ax*qNOR. The BRIL-*Ax*qNOR displays an elongated conformation (Rg = 36.53 Å) compared to the compact native *Ax*qNOR (Rg = 36.17 Å), with substantial variation in pairwise residue’s contact distances at the dimer interface. This is evident from the changes in Cα interatomic distances between equivalent residues in the two structures (Figure 3B).

**Figure 3:**
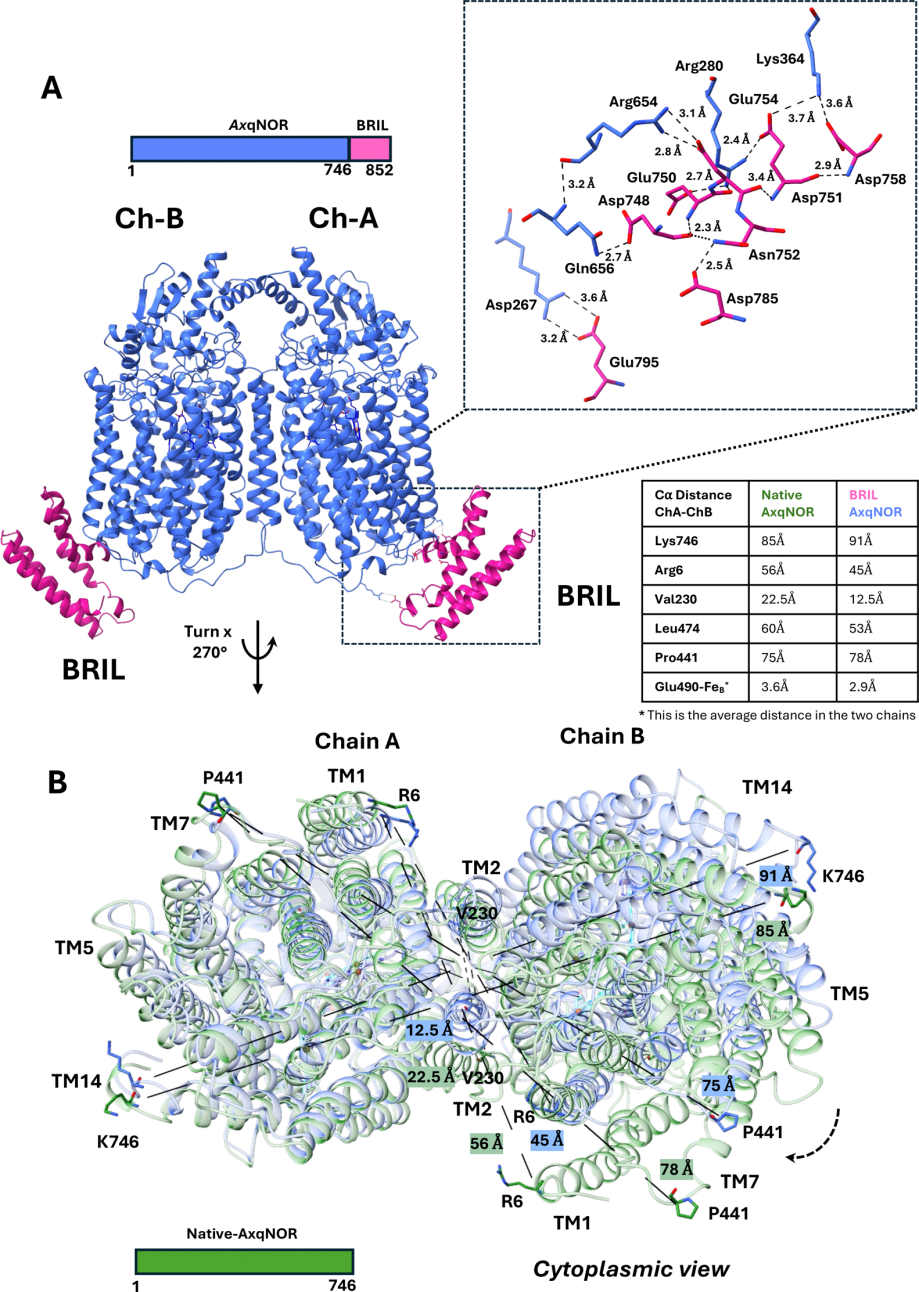
Structural impact of BRIL fusion on *Ax*qNOR. (A) Predicted ionic interactions at the BRIL-C-terminal *Ax*qNOR interface include Lys364-Asp758, Lys364-Glu754, Arg280-Glu754, Arg280-Glu750, Asp751-Arg654, Gln656-Asp748, Arg267-Glu795, and Asp785-Asn752. (B) While the monomeric fold in native *Ax*qNOR and BRIL-*Ax*qNOR shows only subtle changes, the dimeric assembly undergoes pronounced conformational rearrangements. These involve shifts in TM helices, altering Cα distances at equivalent positions in TM1 Arg6, TM2 Val230, TM7 Pro441, and TM14 Lys746, resulting in a distinct dimeric conformation in BRIL-*Ax*qNOR relative to native *Ax*qNOR (see Table S1, also). In the BRIL-*Ax*qNOR construct, qNOR is shown in cornflower blue and BRIL in pink, while the native *Ax*qNOR is shown in green. Domain boundaries are clearly delineated, and Cα distances (Å) at equivalent positions are indicated.

For example, in native *Ax*qNOR the distance between TM1 residues Arg6 (Chain A-Chain B) is more than 10 Å longer than in BRIL-*Ax*qNOR. Similarly, the distance between Lys746 (Chain A-Chain B) near the edge of TM14 is increased by ∼6 Å in BRIL-*Ax*qNOR relative to the native structure. Another pronounced difference is observed in TM2, where the interhelical distance between Val230 (Chain A–Chain B) is reduced by 10 Å in BRIL-*Ax*qNOR compared to native *Ax*qNOR. In addition, the interatomic distance between Leu474 residues in TM8 increases by 7 Å in the native structure, while the Pro441-Pro441 distance in TM7 increases by ∼3 Å in the BRIL fusion relative to the native structure (Figure 3B, Supplementary Table 1). Notably, the helices mediating subunit association display nonconserved contact patterns, resulting in overall conformational changes that suggest the BRIL fusion may perturb the native conformation of *Ax*qNOR.

At the monomeric level, subtle conformational shift is observed in TM1, TM6, TM7, TM9, and TM10, leading to altered side-chain positioning of catalytic residues (e.g., Glu490, Glu494, and Glu559) and their coordination geometry with Fe_B_. For instance, the Glu490-Fe_B_ distance increases from 2.9 Å in the BRIL-*Ax*qNOR to ∼3.3 Å in the native *Ax*qNOR structure. Also, the distance between the heme *b3* position and the nonheme metal Fe_B_ is increased from 3.3 Å in the native structure to 3.9 Å in BRIL-*Ax*qNOR while the coordination with proximal His residues remains conserved (Supplementary Figure 7).

Apart from that, the presence of BRIL appears to have a stabilizing effect on *Ax*qNOR, likely contributing to improved 3D reconstruction. For instance, TM1 and TM7, which are barely visible in the native *Ax*qNOR structure solved at 2.6 Å resolution at pH 8 (∼470k particles), are clearly resolved in the BRIL-*Ax*qNOR structure from the reprocessed data set at 3.4 Å resolution (85k particles, current study), and in the 2.2 Å structure (∼400k particles).^22,23^ This strongly suggests that the improved reconstruction, irrespective of resolution, arises from the BRIL fusion, which may have led to better cryoEM sample preparation and/or induced greater homogeneity, resulting in clear visibility of TM1 and TM7. Interestingly, enzymatic assays revealed a modest difference in activity between native *Ax*qNOR and BRIL-*Ax*qNOR, with the latter showing only around 5% lower activity.^22^ The slight increase in the activity of native *Ax*qNOR may arise from subtle changes in the overall dimeric organization or local helical conformations, which may facilitate substrate binding or product release more efficiently than in the non-BRIL native structure. Structural analyses further reveal subtle changes in the putative substrate entry and product release pathway, which may affect the turnover rate (Supplemental Figure S7).^15,16,22,23^ However, the relatively small change in enzyme activity suggests that, despite these conformational differences, the catalytic site remains largely unaltered, resulting in only a minimal impact on function. This further indicates that enzymatic activity assays alone may not accurately reflect the native quaternary organization.

### Electron Transfer Pathway and the Identification of Quinol Binding Site

A distinct feature of qNORs is their ability to use the membrane-soluble ubiquinol pool as an electron donor, with electrons flowing from periplasmic quinol-binding sites to heme *b*_3_ catalytic redox centers in the protein core *via* heme *b*.^1^

In our earlier work, we proposed a ubiquinol-1 (UQ1) site near heme *b*, based on residual cryoEM density between TM3 and TM14.^23^ This assignment was guided by the *Geobacillus stearothermophilus* qNOR crystal structure (PDB: 3AYG), in which the equivalent pocket accommodates 2-heptyl-hydroxyquinoline N-oxide (HQNO).^16^ However, subsequent *Ax*qNOR data sets revealed that the putative UQ1 density was weak and sporadic, suggesting either partial occupancy or an artifact. By contrast, a reproducible density consistently appeared adjacent to Trp718 on TM14, directly exposed to the putative membrane boundary (Figure 5A). The planar, aromatic headgroup matched that of ubiquinol, but the aliphatic tail merged into the surrounding DDM micelle, hindering model building. We infer that endogenous ubiquinol (likely UQ8) copurifies from *E. coli* membranes, partitions into the detergent micelle, and occupies this surface-exposed entry site.

Notably, the ambiguous “UQ1” density near heme *b* reappeared when *Ax*qNOR was incubated with 5 mM quinol (benzene-1,4-diol or HQE) or hydroxyquinol (benzene-1,2,4-triol or HQN) prior to grid preparation. These short-chain, quinol analogues—lacking an isoprenoid tail—enhanced occupancy and produced stronger density both at the primary quinol binding site **(** *Q*_*o*_ **)** in the outer periplasmic region near Trp718 and the secondary internal site **(** *Q*_*i*_ **)** adjacent to heme *b* within the protein core (Figure 4). This suggests that ligand incubation during sample preparation enhances occupancy, with the second ubiquinol molecule likely entering through the primary Trp718 site and then reaching the secondary binding site adjacent to heme *b*, which may otherwise be lost during protein purification or only sporadically retained, explaining its variable appearance across different 3D reconstructions.

**Figure 4:**
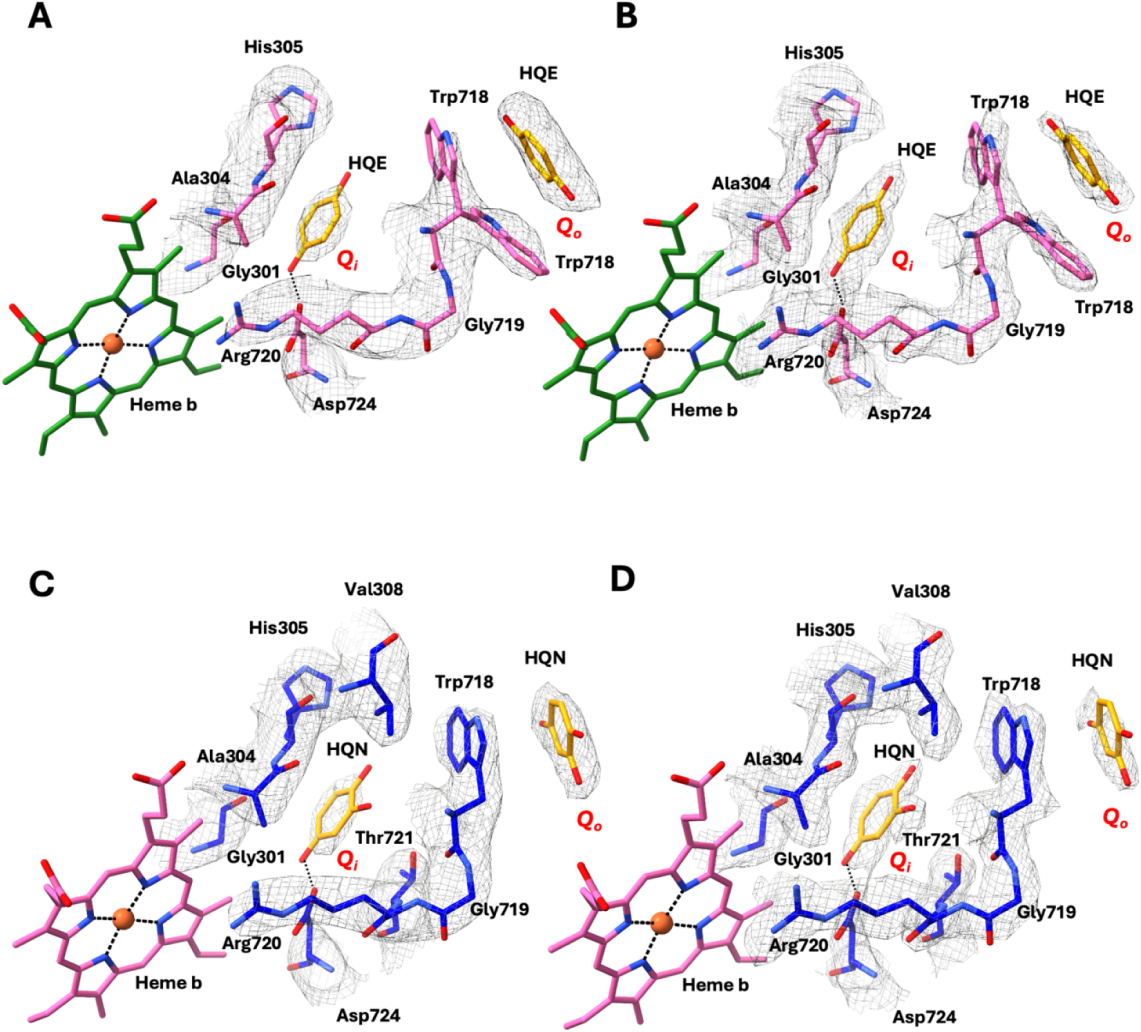
Quinol and hydroxyquinol binding sites in *Ax*qNOR. (A) Schematic model illustrating the Quinol (HQE) interactions at the putative site ***Q_o_*** near Trp718 and ***Q_i_*** site adjacent to low-spin heme *b* in *Ax*qNOR. The corresponding cryoEM density map is shown, highlighting the HQE and interacting side-chain residues. (B) As in panel A, but using the sharpened cryoEM map to emphasize the density quality for HQE and surrounding residues. (C) Schematic model depicting the possible hydroxyquinol (HQN) interactions at the ***Q_o_*** site near Trp718 and the ***Q_i_*** site near low-spin heme *b* in *Ax*qNOR, with the cryoEM density map highlighting the ligand and residue fits. (D) As in panel C, but with the sharpened cryoEM map to underscore the fit of HQN and interacting side chains.

To further test this, we mutated Trp718 to Ala and determined the cryoEM structure of the mutant *Ax*qNOR in the presence and absence of quinol. In the *Ax*qNOR^W718A^ structure, density corresponding to quinol at the *Q*_*o*_ site is no longer observed, and no additional density is detected near *Q*_*i*_. However, upon incubation of the *Ax*qNOR^W718A^ with 5 mM quinol prior to grid freezing, density becomes visible near *Q*_*i*_, while *Q*_*o*_ remains empty (Figure 5). Taken together, these results are consistent with the presence of two putative quinol-binding sites and suggest that Trp718 contributes to quinol binding and likely plays an important role in electron transfer.

**Figure 5:**
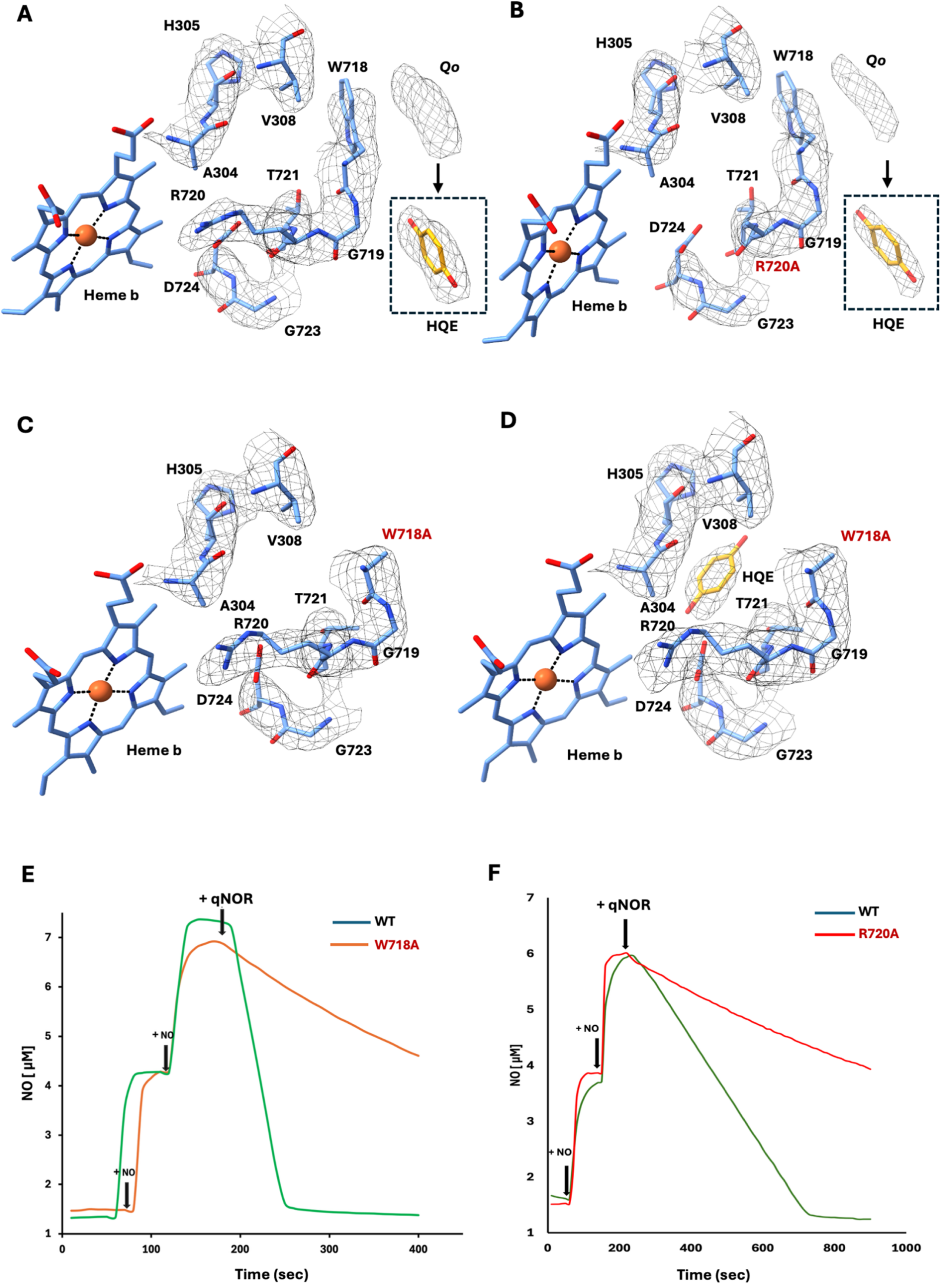
Putative quinol binding at *Q_o_* and *Q_i_* and effects of Trp718/Arg720 mutations on *Ax*qNOR activity. (A) Schematic model showing “ubiquinol-like” density at the ***Q_o_*** site near Trp718 in freshly purified *Ax*qNOR; inset depicts fitting of a quinol molecule. (B) The *Ax*qNOR^R720A^ variant similarly displays density at ***Q_o_*** with quinol precisely fitting into it. (C) *Ax*qNOR^W718A^ shows no density at ***Q_o_*** site. (D) Incubation of *Ax*qNOR^W7218A^ with quinol prior to data collection reveals quinol density at ***Q_i_*** near heme *b*. **(E and F)** Activity measurements show that *Ax*qNOR^W718A^ and *Ax*qNOR^R720A^ variants exhibit decreased NO reduction activity compared to the wild type enzyme.

Retention of endogenous ubiquinol is well documented in membrane proteins solubilized in DDM, and many quinol-oxidizing enzymes are proposed to possess two quinol-binding sites despite their diverse redox chemistries and enzymatic activities.^33–35^ The best-characterized example is *E. coli* cytochrome *bo_3_* ubiquinol oxidase, which functions as a terminal oxidase in aerobic respiration, using ubiquinol as the electron donor to catalyze the four-electron reduction of molecular oxygen to water. Biochemical studies by Sato et al. proposed two ubiquinol-binding sites in cytochrome *bo_3_*: a high-affinity site (Q_H_) and a low-affinity quinone-binding site (Q_L_).^36^ Electrons are proposed to flow from ubiquinol at Q_L_ to a tightly bound quinone at Q_H_, and then to the heme-copper catalytic center, or alternatively, it passes directly from Q_L_ to low-spin heme *b*.

Early biochemical and structural studies confirmed the presence of quinol at Q_H_; in contrast, the proposed Q_L_ site remained elusive. Evidence for Q_L_ largely derives from mutagenesis studies, in which alterations of residues thought to form this site reduced ubiquinol oxidase activity.^37,38^ For example, Ma et al. reported that Tryptophan 136 in subunit II of *E. coli* cytochrome *bo*_3_ may participate in ubiquinol binding near the Q_L_ site.^39^ Despite extensive efforts, no high-resolution structure has captured two ubiquinol molecules simultaneously bound at distinct Q_H_ and Q_L_ sites. Even in the crystal structure of *E. coli* ubiquinol oxidase reported by Li et al. (PDB: 1FFT) only a single ubiquinone molecule was assigned to Q_H_, while Q_L_ was not observed.^40^ More recent biochemical and structural analyses (PDB: 7CUB, 6WTI, and 8QQK) have seriously questioned the existence of Q_L_ altogether, strongly supporting a single functional quinone-binding site Q_H_ and suggesting that any secondary Q_L_ site may be absent.^41–44^

Interestingly, our ligand-bound cryoEM structures of *Ax*qNOR provide structural evidence for two distinct quinol-binding sites, analogous to the dual-site model proposed for cytochrome *bo_3_*, representing the first such observation in any nitric oxide reductase family. In *Ax*qNOR, the Trp718-associated primary site (Q_*o*_) may correspond to the putative Q_L_, while a second site (Q_*i*_), located in a pocket adjacent to heme *b*, represents the putative Q_H_ site. Based on this model, electrons are proposed to transfer from Q_*o*_ and Q_*i*_ to the catalytic heme *b*, supporting a dual-site mechanism of quinol oxidation in NORs that mirrors the classical cyt *bo_3_* paradigm.^36^

Based on the spatial location of the quinol-binding sites, site-directed mutagenesis data, structural similarity to the homologous *Gs*qNOR, and functional analogy to quinol-binding sites in cytochrome *bo*_*3*_ ubiquinol oxidase, we propose that electron transfer from Q_*o*_ and Q_*i*_ to the catalytic heme *b* in *Ax*qNOR may occur via three possible mechanisms:

**(i) Direct** *Q*_*o*_ **→ heme** *b* **transfer**. Tryptophan residues have been shown to play an important role in redox enzymes, where electron transfer (ET) occurs over long distances (>25 Å) *via* tunneling.^45–47^ In the *Ax*qNOR structure, HQE or HQN is bound to Trp718 near the *Q*_*o*_ site through a planar π–π stacking interaction, with its benzene ring positioned approximately 16 Å from heme *b*. This geometry supports a pathway in which electrons delocalize onto Trp718 before being directly transferred to heme *b*.

In this position, Trp718 adopts two conformations and engages in hydrophobic contacts with Gly308 and Val719 (∼4 Å), modestly increasing the available planar surface and potentially enhancing quinol binding and electron tunneling efficiency (Figure 4A). A similar effect has been reported for the *Pseudomonas aeruginosa* azurin mutant Re126WWCu, in which two adjacent tryptophan residues (W124 and W122; indole–indole separation of 3.6–4.1 Å) were approximately 100-fold more effective than a single tryptophan in accelerating electron transfer, supporting long-range electron transport of up to ∼20 Å *via* Trp-mediated hopping.^48^ We evaluated the potential of the Trp718 indole to act as an electron “hopping site” by mapping its spatial relationship to the heme *b*. Trp718 lies at approximately 13 Å from heme *b*, placing it within the range for efficient aromatic-mediated electron tunneling. Alternatively, electron delocalization along this motif (Trp718 → Gly719 → Arg720) could reach heme *b*, which is only ∼3.5 Å from the guanidinium group of Arg720. The positively charged guanidinium is expected to modulate local electrostatics, enhancing the redox potential of heme *b* and facilitating this pathway (Figure 6A).

**Figure 6:**
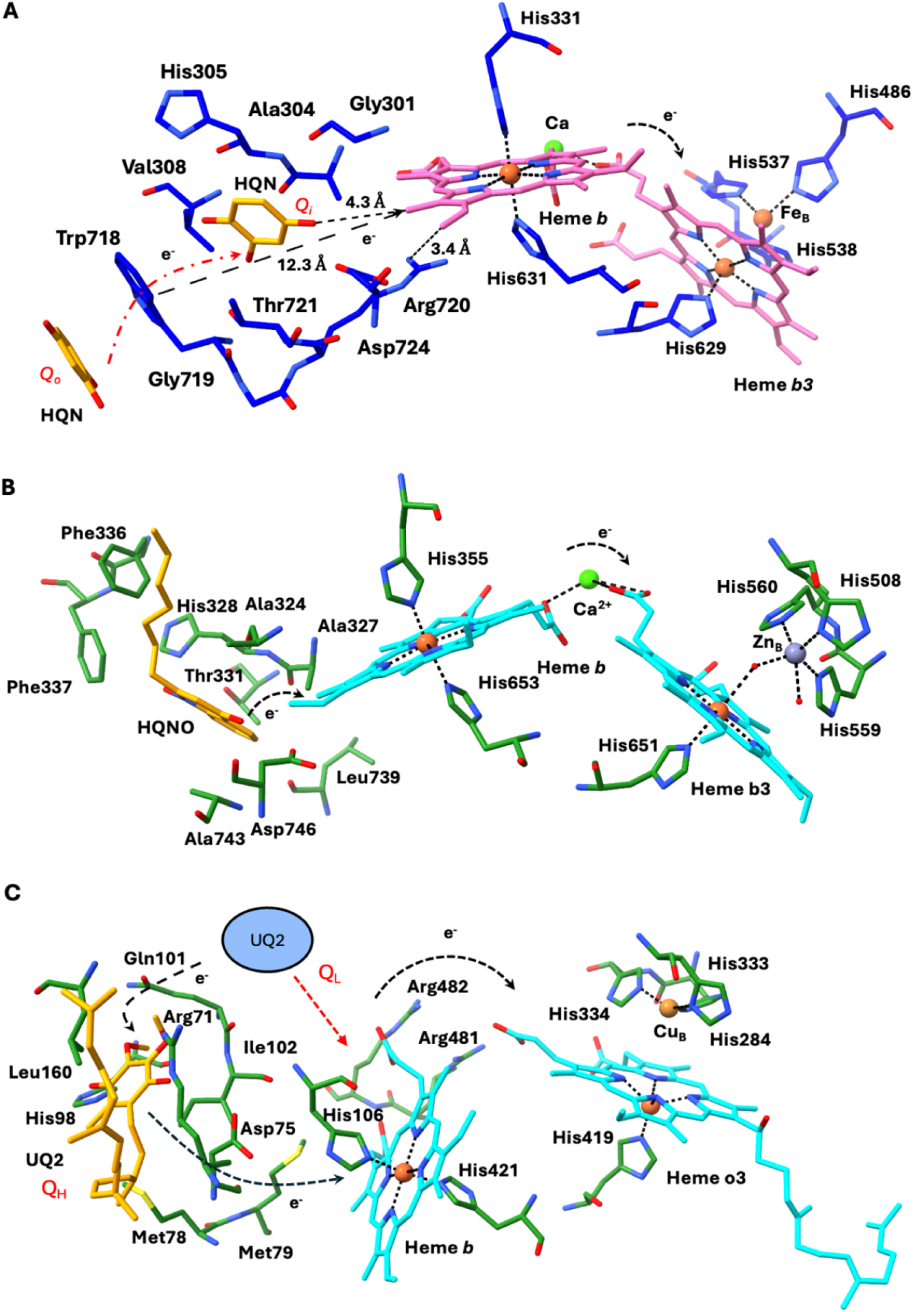
Schematic representation of the ubiquinol mediated electron transfer pathway. (A) Possible ways of electron transfer from the putative quinol (HQE) or hydroxyquinol (HQN) binding site to the binuclear reaction center (heme *b*_3_ and Fe_B_) in *Ax*qNOR. (B) A possible electron path from 2-heptyl-4-hydrxy quinoline N-oxide (HQNO) to the binuclear center (heme *b*_3_ and Zn_B_) in *Gs*qNOR (PDB 3AYG). (C) A possible electron path from ubiquinone-2 (UQ2) or ubiquinone-8 (UQ8) to the binuclear reaction center (heme *o*_3_ and Cu_B_) in *E. coli* ubiquinol oxidase (PDB 7CUB).

Consistent with the above findings, *Ax*qNOR^W718A^ and *Ax*qNOR^R720A^ variants showed an approximately 85% loss of enzymatic activity, highlighting the functional importance of Trp718 and Arg720 in catalysis (Figure 5E and 5F).

**(ii) Direct** *Q*_*i*_ **→ heme** *b* **transfer**. In *Ax*qNOR, the Q_*i*_ site lies approximately 4.5 Å from heme *b.* At this position, the quinol analogue HQE/HQN occupies a hydrophobic pocket formed by Gly301, Ala304, and Val308 and engages in hydrogen-bond interactions with His305 and Asp724, residues that are conserved within the qNORs but absent in cNORs. In contrast, Trp718 and Arg720 are conserved in both qNORs and cNORs, suggesting a shared element of the broader electron-transfer framework that predates the evolutionary divergence of qNORs from cNORs (Figure 7 and Supplemental Figure 8). The proximity between Q_*i*_ and heme *b* makes it compatible with direct electron transfer, as previously proposed for *GsqNOR* by Matsumoto *et al*.^16^ *Gs*qNOR is a notable exception in which Trp718 is replaced by the nonaromatic residue Leu740. As a result, the quinol analogue HQNO binds deeper and much closer (∼4 Å) to heme *b*, positioning it within an ideal distance for direct electron transfer (Figure 6A and 6B).^16^ Consistent with these structural observations, biochemical characterization of a qNOR from *Persephonella marina* by Sheraden identified a quinol-binding site involving the homologous residues His295, Arg705, and Asp709.^49^ Mutation of these residues resulted in a complete loss of enzymatic activity, further supporting a conserved functional role for the Q_*i*_ site in mediating direct electron transfer to heme *b* in the qNOR family.^49^

**Figure 7:**
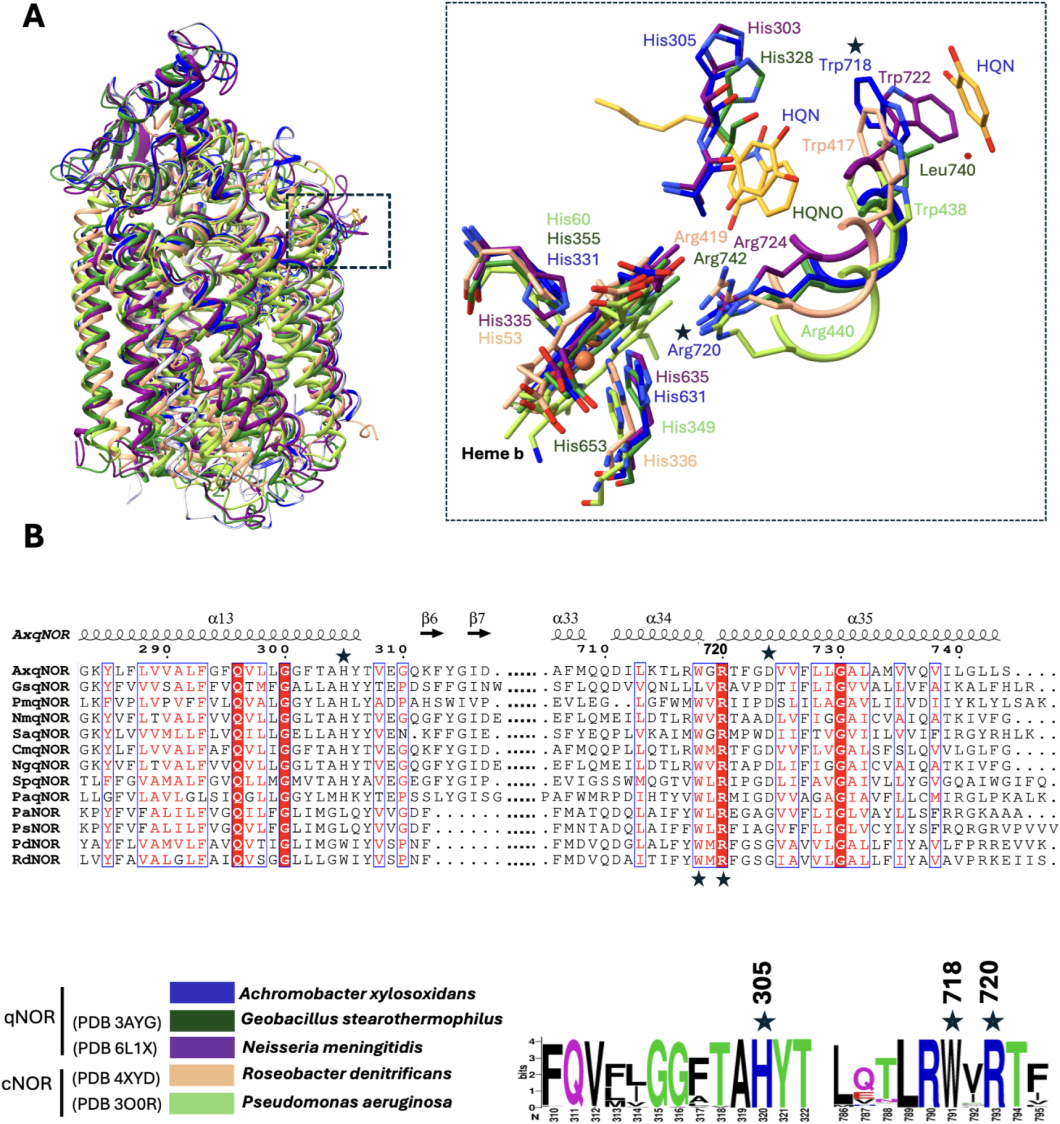
Structural comparison of quinol-binding sites in NORs. (A) Structural superposition of *Ax*qNOR bound to HQE, *Gs*qNOR bound to HQNO, and *Nm*qNOR, *Pa*cNOR, and *Rd*cNOR in the apo state. Individual structures are color-coded as indicated, with corresponding PDB IDs shown. (B) Multiple sequence alignment highlighting the conservation and variability of residues within the quinol-binding pocket. Conserved and semiconserved residues are indicated, including key positions such as Trp718 and Arg720. WebLogo representation showing the conservation and variability of amino acids surrounding the quinol-binding region derived from clustered qNOR sequences (nr_cluster_seq). The qNOR sequences shown in the alignment are from *Achromobacter xylosoxidans*, *Geobacillus stearothermophilus*, *Persephonella marina*, *Neisseria meningitidis*, *Staphylococcus aureus*, *Cupriavidus metallidurans*, *Neisseria gonorrheae*, *Synechocystis sp*., and *Pyrobaculum aerophilum*, whereas cNOR sequences are from *Roseobacter denitrificans*, *Pseudomonas aeruginosa*, *Pseudomonas denitrificans*, and *Pseudomonas stutzeri*.

**(iii) Sequential** *Q*_*o*_ **→** *Q*_*i*_ **→ heme** *b* **transfer**. We propose a third electron-transfer pathway in *Ax*qNOR that is analogous to the sequential pathway described for cytochrome *bo*_3_ ubiquinol oxidase. In *Ax*qNOR, two quinol molecules associated with the Q_*o*_ and Q_*i*_ sites are separated by approximately 10 Å. Based on their spatial arrangement, the chemical environment of the surrounding residues, and analogy to cytochrome *bo*_3_, we propose a sequential electron-transfer pathway in which electrons are relayed from Q_*o*_ → Q_*i*_ → heme *b*, with the tightly bound quinol at the Q_*o*_ site potentially mediating this process (Figure 6A and 6C).^36^ Such a cooperative transfer mechanism is expected to facilitate efficient coupling between quinol oxidation and reduction of the heme *b*-heme *b*_*3*_-Fe_B_ catalytic center during NO reduction.

Pairwise sequence and structural alignments of *Ax*qNOR with homologous NORs reveal that Trp718 and Arg720 are highly conserved, suggesting a similar putative electron donation pathway across qNORs, including *Neisseria meningitidis* (Figure 7).

Structural comparison of *Ax*qNOR (as purified at pH 6.5) with *Ax*qNOR bound to HQE or HQN revealed no major changes in the overall structure (rmsd ∼ 0.4 Å) and insignificant changes in the active sites (heme *b*_*3*_-Fe_B_ distance: ∼3.5 Å in the as-purified sample versus ∼3.2 Å in HQE/HQN-bound *Ax*qNOR) (Supplementary Figure S9). This minimal change may be due, in part, to the as-purified sample already containing a partially occupied ubiquinol molecule near Trp718 at ***Q***_***o***_ while ***Q***_***i***_ remains empty, or because HQE/HQN binding has little impact on the active site geometry.

### pH-Dependent Enzymatic Activity of *Ax*qNOR

The *Ax*qNOR enzyme exhibited a distinct bell-shaped activity profile, with optimal NO reduction at pH 6.5 and a marked decrease in activity at both pH 5.5 and pH 8.0 (Supplementary Figure S10). This activity pattern likely relates to the p*K*_a_ values of key active-site residues, particularly the histidine residues ligating with heme *b* (His331 and His631) and heme *b3* (His486, His537, His538, and His629) and glutamate residues in the vicinity of the active site (Glu490, Glu494, and Glu559) forming the proton-transfer network. These residues are critically involved in proton delivery in NORs, facilitating proton-coupled electron transfer (PCET) that is essential for stabilizing reaction intermediates and enabling efficient catalysis.^18,31,50^ At pH 6.5, which lies close to the imidazole side-chain p*K*_a_ of histidine (∼6.0), the coordinating histidine is partially protonated. This partial protonation introduces a positive electrostatic influence that would raise the redox potential of the binuclear heme center, thereby facilitating electron acceptance from the quinol donor. Simultaneously, the glutamate–water network is sufficiently protonated to provide an efficient pathway for proton delivery to the active site. The synchronized alignment of favorable electron transfer and proton availability results in the highest catalytic efficiency at this pH. In contrast, at pH 8.0, the histidine is predominantly deprotonated, presumably lowering the heme’s redox potential and making electron transfer less favorable, while the glutamate residues are also largely deprotonated, diminishing proton supply to the active site. These combined effects slow down proton-coupled electron transfer and reduce NO reduction activity. On the acidic side, at pH 5.5, histidine and glutamate residues are excessively protonated, which disrupts the balance of proton-coupled electron transfer. Moreover, being in an overly acidic environment can perturb the global stability of the protein, affecting its fold and dynamics, and thereby further compromising activity. To examine pH-dependent structural changes, we solved the native *Ax*qNOR structure at pH 6.5. Superposition with the pH 8.0 structure revealed no notable conformational differences (r.m.s.d. ∼0.5 Å), except for the poorly resolved densities of TM1, TM7, and a subtle movement in TM8 at pH 6.5 (Supplementary Figure S10), suggesting structural flexibility that may affect enzymatic activity. The corresponding cryoEM density map of the active site demonstrates preservation of the binuclear center at both pH 6.5 and pH 8.0, with the distance between heme *b*_3_ and Fe_B_ shifting only slightly from 3.5 Å at pH 6.5 to 3.3 Å at pH 8.0. These differences raise the possibility that, at pH 6.5, the slightly expanded spacing may permit better accommodation of the substrate molecules (NO), whereas the more constrained geometry at pH 8.0 could reduce substrate accessibility and contribute to diminished activity. However, the current cryoEM structures alone may not fully explain why *Ax*qNOR exhibits higher activity at pH 6.5.

The pH-dependence of *Nm*qNOR that used a variety of buffering agents to cover the pH range, showed again, a bell-shaped curve with a pH-optimum of 7.5. In contrast, the pH dependence of cNOR from *P. aeruginosa* over a pH range of 6 to 10, using different buffers, did not show a bell-shaped curve but showed that *cNOR* is more active under acidic conditions (pH 6.0) than under alkaline conditions (pH 9.0).^51^ This may reflect a genuine difference between the two types of NORs, cNOR and qNOR.

## Conclusion

The cryoEM structure of full-length native quinol-dependent nitric oxide reductases (qNOR) determined here represents the first structure of native qNOR without truncation and fusion with BRIL. The high-resolution structure of native qNOR reveals a dimeric assembly that is stabilized by extensive interaction, particularly between residues of TM2 of each monomer. The catalytic core is clearly defined, with all the ligands provided by helices 4, 8, 9, and 12, with hemes *b* and *b_3_* positioned in place by a calcium ion. The iron of the heme *b_3_* ligates to H629 from TM12 on one side of the heme and with nonheme Fe *via* the μ-oxo bridge that is ligated to H486 from TM8. We provide the pH-dependence of the enzymatic activity by deploying the MMH buffer, which allows effective buffering capacity over the whole pH range (pH 5.5 to 8.0), revealing a clear bell-shaped curve with optimum activity at pH 6.5. A comparison of the structures determined at pH 6.5 and pH 8 shows close similarity, except for greater flexibility of TM1 and TM7 at pH 6.5 and a marginal increase in the distance between the heme *b_3_* and Fe_B_ metal centers at pH 6.5, an expansion which may permit accommodation of two NO molecules and a higher rate of turnover. We were able to identify putative ubiquinol binding sites from structures determined by incubating native *Ax*qNOR with the ubiquinol analogues, quinol (HQE) or hydroxyquinol (HQN), with clear density near Trp718 (***Q***_***o***_) as well as in a pocket adjacent to heme *b* (***Q***_***i***_). We suggest that in *Ax*qNOR, electron flow is mediated by tryptophan. The two binding sites share a parallel with *E. coli* ubiquinol oxidase, with a conserved electron transfer pathway in the quinol-dependent members of the respiratory heme-copper oxidase superfamily.

The determination of the cryoEM structure of native *Ax*qNOR has allowed us to assess the impact of BRIL by comparing the cryoEM structures of native *Ax*qNOR with BRIL-*Ax*qNOR. Despite a very similar level of enzymatic activity, the dimeric assembly exhibits pronounced conformational differences, influencing intersubunit packing and altering the overall protein architecture, as reflected in the total buried area. For native *Ax*qNOR, it is 15,917 Å^2^ compared to 23,031 Å^2^ in BRIL-*Ax*qNOR. Also, the distance between TM1 residues Arg6 (Chain A-Chain B) in the native *Ax*qNOR structure is more than 10 Å longer compared to the distance in BRIL-*Ax*qNOR. Another pronounced difference is observed in TM2, where the interhelical distance between Val230 (Chain A-Chain B) is reduced by 10 Å in BRIL-*Ax*qNOR compared to native *Ax*qNOR. This opens a wider question regarding the integrity of the structures when a protein is tagged with a reporter protein such as Green Fluorescence Protein (GFP) or fused with BRIL, which not only increases the molecular size but also serves as a widely used fiducial marker, providing distinguishable features that facilitate image alignment and structure determination.^52^

## Materials and Methods

### Expression and Purification of wild type Native *Ax*qNOR and its Arg720Ala and Trp718Ala Variants

The wild-type native *Ax* qNOR and its R720A and Trp718A mutant plasmids were transformed and expressed in *E. coli* strain C41 (DE3). A single colony carrying the recombinant plasmid was inoculated into 2 × YT media and grown overnight at 37 °C, then diluted into 500 mL of 2 × YT media in a baffled flask supplemented with 50 μg·mL^–1^ kanamycin and grown to an OD_600_ of 2. At this stage, cultures were supplemented with 0.2 mM FeCl_3_ and 0.2 mM 5-aminolevulinic acid (5-ALA), and protein expression was induced with 500 μM IPTG. Cultures were then grown overnight at 18 °C, harvested, and washed in 50 mM Tris-HCl (pH 8.0) containing 150 mM NaCl.

Cell pellets were resuspended in lysis buffer (50 mM Tris-HCl pH 8.0, 150 mM NaCl, 2 mM EDTA, 2.5 mM MgCl_2_, 1 mg/mL lysozyme, and protease inhibitors) and lysed by sonication. Cell debris was removed by centrifugation, and membranes were isolated by ultracentrifugation and resuspended in 50 mM Tris-HCl, 150 mM NaCl. Membranes (7 mg/mL) were solubilized in 1% β-DDM for 2 h, and insoluble material was removed by centrifugation. The solubilized fraction was incubated with Ni-NTA resin, washed with 20 mM and 35 mM imidazole, and eluted with 150 mM imidazole. Protein-containing fractions were pooled based on SDS-PAGE and UV–vis absorbance (A_410_/A_280_ > 0.6), concentrated, and further purified by size-exclusion chromatography (HiLoad Superdex 200 16/60 pg) in 25 mM Tris-HCl pH 8.0, 150 mM NaCl, 0.05% DTM. For protein purification at pH 6.5, the size-exclusion buffer was modified to 25 mM Mes pH 6.5, 150 mM NaCl, 0.05% DTM. Dimeric fractions were run and analyzed by SDS-PAGE for purity, and those with A_410_/A_280_ > 0.7 were pooled, concentrated to ∼10 mg/mL, flash-frozen in liquid nitrogen, and stored at −80 °C for biochemical and structural studies.

### Activity Measurement

qNOR activity was measured using a Clark-type electrode connected to an ISO-NO Mark II system (World Precision Instruments). Assay components were made anaerobic by flushing the reaction vials with N_2_. The reaction buffer contained 50 mM sodium citrate (pH 6.0), 0.05% DTM or β-DDM, 100 mM d-glucose, 10 μg/mL glucose oxidase, and 10 μg/mL catalase. Electron donation was provided by 10 mM sodium ascorbate and 100 μM phenazine methosulfate (PMS). Nitric oxide (NO) was supplied by the addition of a 2 mM saturated NO aqueous solution prepared in 50 mM Tris-HCl (pH 7.0), added to a final concentration of 20 μM. NO consumption was initiated by the addition of purified protein. For pH dependence, the standard citrate buffer was replaced with 75 mM MMH buffer (25 mM MES, 25 mM malonic acid, and 25 mM HEPES, pH 5.5–8.0), which allowed buffering activity over the desired pH range.

### CryoEM Sample Preparation, Data Collection, and Image Processing

Native *Ax*qNOR was diluted to 5 mg mL^–1^, and 3.5 μL aliquots were applied to glow-discharged Quantifoil Au R1.2/1.3 (or Quantifoil Cu R1.2/1.3) holey carbon grids. For ligand-binding data sets, the protein was incubated with 5 mM quinol or hydroxyquinol prior to vitrification. Grids were plunge-frozen in liquid ethane using a Vitrobot Mark IV (FEI) with a 10-s wait time, followed by blotting for 6 s, at a blot force of 1, under conditions of 100% humidity and 4 °C. Data collection was performed on a Titan Krios G2 electron microscope operated at 300 kV with an X-FEG source, and movies were recorded in EPU v3.10 using a Falcon 4i detector with a Selectris energy filter at 165 k× magnification, corresponding to a pixel size of 0.74 Å. For BRIL-*Ax*qNOR data, see Gopalasingam et al. for more details.^22^ Additional experimental details, together with cryoEM data processing workflows for all *Ax*qNOR data sets presented in this manuscript, are provided in Supplementary Tables 2–6 and illustrated in Supplementary Figures 11–19 (Supporting Information).

### Model Building and Refinement of CryoEM Structures

Model building of native *Ax*qNOR at pH 8.0, pH 6.5, and ligand-bound *Ax*qNOR began with rigid-body fitting of individual chains from the high-resolution dimeric *Ax*qNOR structure (PDB: 8BGW) into their respective cryoEM density maps using ChimeraX.^53^ Subsequent refinement was carried out with iSOLDE, followed by iterative cycles of manual rebuilding in Coot and real-space refinement in Phenix with geometric constraints to accurately position the ligands, heme *b*, heme *b*_3_, Fe_B_, Ca^2+^ ion, and water molecules, as well as to model any missing regions of the protein chains.^54–56^ For accurate HQE modeling, σ thresholds of 6.4 (for the original map) and σ 5.4 (for the sharpened map) were applied in Coot.^56^ For HQN, the corresponding thresholds were set at σ 7.5 and σ 5.7, respectively. These σ levels effectively removed background detergent noise, enabling confident model building. Model building of BRIL-*Ax*qNOR was initiated by rigid-body fitting of the best AlphaFold3 model (model #AF3) into the cryoEM map using ChimeraX, followed by refinement in iSOLDE and *phenix.real_space*.^53–55,57^ Stereochemical quality was assessed with Coot and MolProbity.^56,58^ Comprehensive model validation was performed using the Phenix validation module, and figures were prepared with ChimeraX.^53,55^

## Supplementary Material



## Data Availability

The data that support the findings of this study are available from the corresponding author upon reasonable request.

## Notes

Additional experimental details, cryoEM data processing workflow, data collection and refinement statistics, EM maps; structural analysis, AlphaFold3 models (Supplementary Table 1); Cα distances at equivalent positions in native *Ax*qNOR and BRIL-*Ax*qNOR (Supplementary Tables 2, 3, 4, 5, and 6); cryoEM data collection, refinement and validation statistics (Supplementary Figures S1 to S10); detailed presentations of key structural elements in a variety of structures presented in the manuscript (Supplementary Figures S11 to S19) (PDF)
