# Supplementary material for: CryoEM Structures of Native Quinol-Dependent Nitric Oxide Reductase in Resting and Quinol-Bound States
Source: ACS Bio Med Chem Au. 2026 Mar 13;6(2):145–59. doi: 10.1021/acsbiomedchemau.5c00245 (PMC13087809; doi:10.1021/acsbiomedchemau.5c00245)
Supplement: Supplementary file 1 [file bg5c00245_si_001.pdf]

Supporting Information

CryoEM structures of native quinol-dependent Nitric Oxide Reductase in resting and quinol bound states

Faisal T. Khaja[1], Allegra Mboukou[1], Louie P. Aspinall[2,3], Charlotte E. Hawksworth[2,3], Robert R. Eady[1], Svetlana V. Antonyuk[1]*, Stephen P. Muench[3,4]* and S. Samar Hasnain[1]*

[1]Department of Biochemistry, Cell and Systems Biology, Institute of Systems, Molecular and Integrative Biology, University of Liverpool, Liverpool, L69 7ZB, UK

[2]School of Molecular and Cellular Biology, Faculty of Biological Sciences, University of Leeds, Leeds, LS2 9JT, UK

[3]Astbury Centre for Structural Molecular Biology, University of Leeds, Leeds, LS2 9JT, UK

[4] School of Biomedical Sciences, Faculty of Biological Sciences, University of Leeds, Leeds, LS2 9JT, UK

*Corresponding authors: S.P.Muench@leeds.ac.uk, S.Antonyuk@liverpool.ac.uk, s.s.hasnain@liverpool.ac.uk

Supplementary Materials

Supplemental Table 1: Cα distances at equivalent positions in native *Ax*qNOR and BRIL-*Ax*qNOR

**Cα Distance**	**Native *Ax*qNOR**	**BRIL-*Ax*qNOR**
ChA:Lys746- ChB:Lys746	85 Å	91Å
ChA:Arg6- ChB:Arg6	56 Å	45 Å
ChA:Val230-ChB:Val230	22.5 Å	12.5 Å
ChA:Leu474- ChB:Leu474	60 Å	53 Å
ChA:Pro441- ChB:Pro441	75 Å	78 Å
Glu490-Fe_B	3.6 Å	2.9 Å

Supplementary Table 2: CryoEM data collection, refinement and validation statistics

Name	Native-*Axq*NOR pH8.0	Native-*Axq*NOR pH8.0
**Data Accession**		
PDB	28PN	9WYK
EMDB	56718	66367
**Data Collection**		
Microscope	FEI Titan Krios	FEI Titan Krios
Voltage (kV)	300	300
Detector	Falcon 4i	Falcon 4i
Energy filter slit width (eV)	10	10
Nominal magnification	165k	165k
Software	EPU version 3.10	EPU version 3.10
Pixel size (Å/pixel)	0.74	0.74
Defocus range (μm)	-0.7 to -2.7	-0.6 to -2.4
Exposure time (s)	2.63	2.67
Frames	44	40
Exposure rate (e^{-} $pixel^{-1}$ s^{-1})	8.42	7.25
Electron exposure (e^{-}/$Å^2$)	40.7	51.6
Dose per frame (e^{-}/$Å^2$)	0.924	1.29
Micrographs collected	2,702	7,488
Grid type	Quantifoil Au R1.2/1.3	Quantifoil Cu R1.2/1.3
**Reconstruction**		
Software	CryoSPARC 4.6.0	RELION 4.0.2/ CryoSPARC 4.6.0
Particles used in refinement	91,995	4,73,565
Symmetry	C2	C2
Map resolution when FSC=0.143, 0.5 (masked) (Å)	2.7, 2.8	2.6, 2.9
Map sharpening B-factor ($Å^2$)	-93	-136
**Model Building and Refinement**		
Software	Phenix, CCPEM, Chimera, ChimeraX, Coot	Phenix, CCPEM, Chimera, ChimeraX, Coot
Non-hydrogen atoms	11833	12241
Protein residues	1400	1500
Ligands	8	14
Water	550	114
Average B factors ($Å^2$)		
Protein	59	147
Ligands and water	33 and 72	138 and 104
R.M.S. deviations		
Bond length (Å)	0.005	0.005
Bond angle (º)	1.065	1.021
Ramachandran statistics (%)		
Outliers	0.29	0.33
Allowed	3.95	3.54
Favoured	95.76	96.12
MolProbity score	1.79	1.66
ClashScore	6.49	6.56
Poor rotamers (%)	1.54	1.11
Model vs. Map CC (mask)	0.89	0.88

Supplementary Table 3: CryoEM data collection, refinement and validation statistics

**Name**	**Native-*Axq*NOR pH6.5**	**Native-*Axq*NOR^{R720A} pH6.5**
**Data Accession**		
PDB	9ST9	28PP
EMDB	55213	56720
**Data Collection**		
Microscope	FEI Titan Krios	FEI Titan Krios
Voltage (kV)	300	300
Detector	Falcon 4i	Falcon 4i
Energy filter slit width (eV)	10	10
Nominal magnification	165k	165k
Software	EPU version 3.10	EPU version 3.10
Pixel size (Å/pixel)	0.74	0.74
Defocus range (µm)	-0.6 to -2.4	-0.7 to -2.7
Exposure time (s)	2.31	2.63
Frames	40	44
Exposure rate (e^{-} $pixel^{-1}$ s^{-1})	7.25	8.42
Electron exposure ($e^{-}/Å^{2}$)	40.8	40.7
Dose per frame ($e^{-}/Å^{2}$)	1.02	0.925
Micrographs collected	12,825	10,001
Grid type	Quantifoil Au R1.2/1.3	Quantifoil Au R1.2/1.3
**Reconstruction**		
Software	CryoSPARC 4.6.0	CryoSPARC 4.6.0
Particles used in refinement	149,996	168,294
Symmetry	C2	C2
Map resolution when FSC=0.143, 0.5 (masked) (Å)	3.0, 3.2	2.8, 3.0
Map sharpening B-factor ($Å^{2}$)	-127	-115
**Model Building and Refinement**		
Software	Phenix, CCPEM, Chimera, ChimeraX, Coot	Phenix, CCPEM, Chimera, ChimeraX, Coot
Non-hydrogen atoms	11287	11627
Protein residues	1401	1401
Ligands	8	8
Water	2	354
Average B factors ($Å^{2}$)		
Protein	157	73
Ligands and water	130 and 151	48 and 85
R.M.S. deviations		
Bond length (Å)	0.008	0.006
Bond angle (º)	1.040	1.063
Ramachandran statistics (%)		
Outliers	0.36	0.07
Allowed	3.73	3.73
Favoured	95.91	96.20
MolProbity score	1.83	1.75
ClashScore	4.88	5.74
Poor rotamers (%)	2.44	1.72
Model vs. Map CC (mask)	0.83	0.89

Supplementary Table 4: Cryo-M data collection, refinement and validation statistics

**Name**	**BRIL-*Axq*NOR**
**Data Accession**	
PDB	9WYL
EMDB	66368
**Data Collection**	
Microscope	FEI Titan Krios
Voltage (kV)	300
Detector	K2
Nominal magnification	75,000x
Software	EPU
Pixel size (Å/pixel)	1.07
Defocus range (µm)	-1.5 to -3.5
Exposure time per frame (s)	0.3
Frames	40
Exposure rate (e^- $pixel^{-1}$ s^{-1})	6.21
Electron exposure ($e^-/Å^2$)	65
Dose per frame ($e^-/Å^2$)	1.63
Micrographs collected	3213
Grid type	Quantifoil Au R1.2/1.3
**Reconstruction**	
Software	RELION 4.0.2/ CryoSPARC 4.6.0
Particles used in refinement	100,494
Symmetry	C2
Map resolution when FSC=0.143, 0.5 (masked) (Å)	3.4, 3.5
Map sharpening B-factor ($Å^2$)	-147
**Model Building and Refinement**	
Software	Phenix, CCPEM, Chimera, ChimeraX, Coot
Non-hydrogen atoms	13612
Protein residues	1704
Ligands	8
Water	0
Average B factors ($Å^2$)	
Protein	188
Ligands and water	112
R.M.S. deviations	
Bond length (Å)	0.005
Bond angle (Å)	0.975
Ramachandran statistics (%)	
Outliers	0.0
Allowed	2.71
Favoured	97.29
MolProbity score	1.32
ClashScore	4.07
Poor rotamers (%)	0.60
Model vs. Map CC (mask)	0.83

Supplementary Table 5: CryoEM data collection, refinement and validation statistics

Name	Native-*Axq*NOR pH6.5+HQN	Native-*Axq*NOR pH6.5+HQE
**Data Accession**		
PDB	9WYM	9STA
EMDB	66369	55214
**Data Collection**		
Microscope	FEI Titan Krios	FEI Titan Krios
Voltage (kV)	300	300
Detector	Falcon 4i	Falcon 4i
Energy filter slit width (eV)	10	10
Nominal magnification	165k	165k
Software	EPU version 3.10	EPU version 3.10
Pixel size (Å/pixel)	0.74	0.74
Defocus range (µm)	-0.7 to -2.7	-0.7 to -2.7
Exposure time (s)	2.67	2.67
Frames	31	31
Exposure rate (e^- $pixel^{-1}$ s^{-1})	8.29	8.29
Electron exposure ($e^-/Å^2$)	40.4	40.4
Dose per frame ($e^-/Å^2$)	1.30	1.30
Micrographs collected	23,685	18,744
Grid type	Quantifoil Au R1.2/1.3	Quantifoil Au R1.2/1.3
**Reconstruction**		
Software	CryoSPARC 4.6.0	CryoSPARC 4.6.0
Particles used in refinement	755,700	530,497
Symmetry	C2	C2
Map resolution when FSC=0.143, 0.5 (masked) (Å)	2.3, 2.5	2.4, 2.5
Map sharpening B-factor ($Å^2$)	-94.5	-105.5
**Model Building and Refinement**		
Software	Phenix, CCPEM, Chimera, ChimeraX, Coot	Phenix, CCPEM, Chimera, ChimeraX, Coot
Non-hydrogen atoms	12181	11969
Protein residues	1433	1421
Ligands	16	16
Water	506	354
Average B factors ($Å^2$)		
Protein	97	56
Ligands and water	102 and 82	46 and 53
R.M.S. deviations		
Bond length (Å)	0.009	0.004
Bond angle (°)	1.131	0.997
Ramachandran statistics (%)		
Outliers	0.28	0.0
Allowed	3.72	2.69
Favoured	96.0	97.31
MolProbity score	1.88	1.42
ClashScore	7.30	5.35
Poor rotamers (%)	1.87	0.80
Model vs. Map CC (mask)	0.92	0.88

Supplementary Table 6: CryoEM data collection, refinement and validation statistics

Name	Native-*Axq*NOR^{W718A} pH6.5	Native-*Axq*NOR^{W718A} pH6.5+HQE
**Data Accession**		
PDB	28PQ	28PR
EMDB	56721	56722
**Data Collection**		
Microscope	FEI Titan Krios	FEI Titan Krios
Voltage (kV)	300	300
Detector	Falcon 4i	Falcon 4i
Energy filter slit width (eV)	10	10
Nominal magnification	165k	165k
Software	EPU version 3.10	EPU version 3.10
Pixel size (Å/pixel)	0.74	0.74
Defocus range (µm)	-0.7 to -2.7	-0.7 to -2.7
Exposure time (s)	2.63	2.63
Frames	30	30
Exposure rate (e^- pixel^{-1} s^{-1})	8.42	8.42
Electron exposure (e^-/Å^2)	40.4	40.4
Dose per frame (e^-/Å^2)	1.34	1.34
Micrographs collected	12,938	17,745
Grid type	Quantifoil Au R1.2/1.3	Quantifoil Au R1.2/1.3
**Reconstruction**		
Software	CryoSPARC 4.6.0	CryoSPARC 4.6.0
Particles used in refinement	516,877	651,319
Symmetry	C2	C2
Map resolution when FSC=0.143, 0.5 (masked) (Å)	2.3, 2.4	2.4, 2.4
Map sharpening B-factor (Å^2)	-90.8	-95.6
**Model Building and Refinement**		
Software	Phenix, CCPEM, Chimera, ChimeraX, Coot	Phenix, CCPEM, Chimera, ChimeraX, Coot
Non-hydrogen atoms	12355	12389
Protein residues	1421	1421
Ligands	12	14
Water	818	856
Average B factors (Å^2)		
Protein	47.39	49.21
Ligands and water	35.67 and 59.08	35.9 and 61.7
R.M.S. deviations		
Bond length (Å)	0.004	0.004
Bond angle (Å)	0.976	0.992
Ramachandran statistics (%)		
Outliers	0.21	0.21
Allowed	2.97	3.04
Favoured	96.82	96.74
MolProbity score	1.53	1.59
ClashScore	4.98	4.24
Poor rotamers (%)	1.26	1.71
Model vs. Map CC (mask)	0.89	0.89

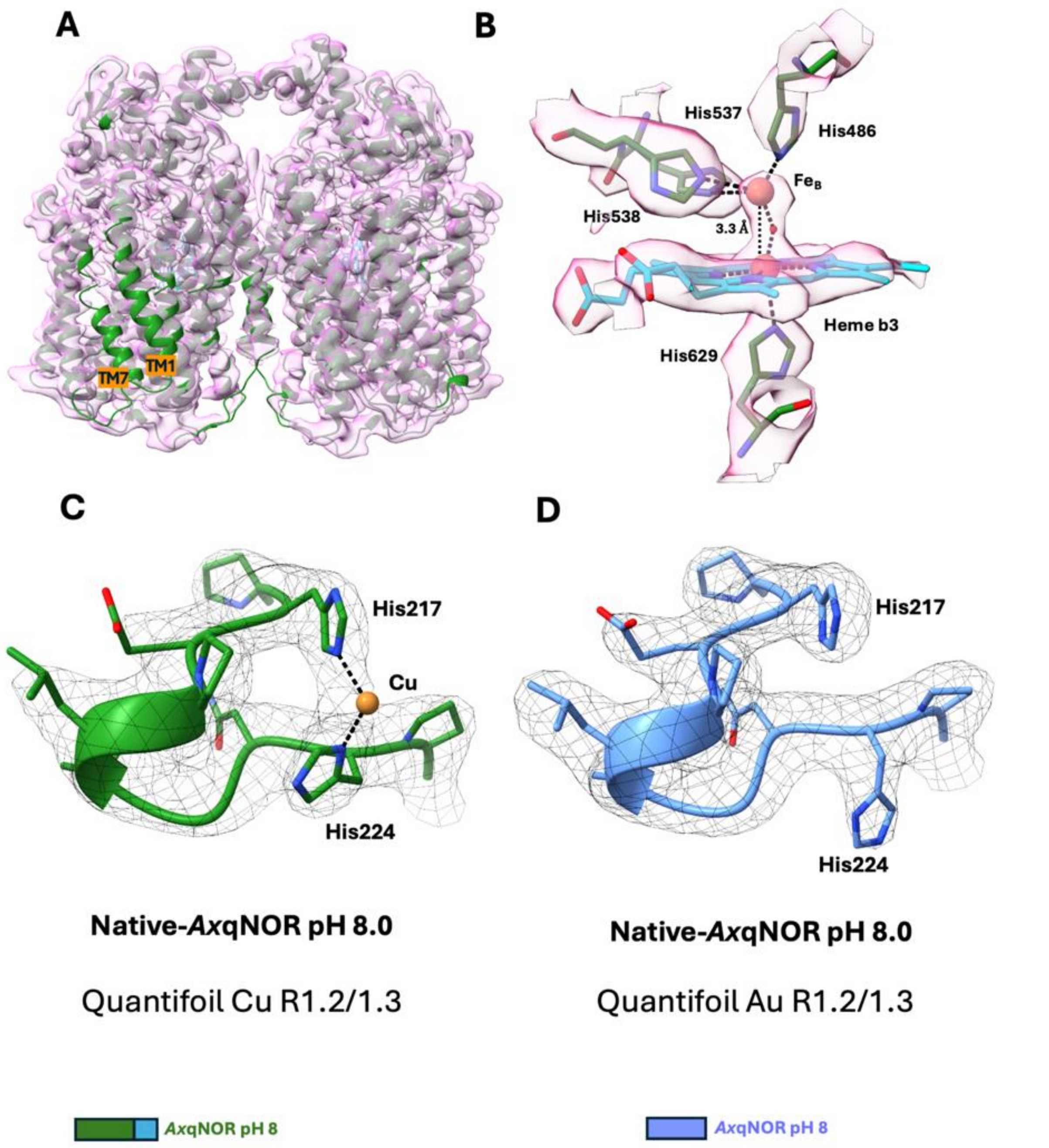

Supplementary Figure 1 | Structure and features of native *Ax*qNOR at pH 8.0. (A) CryoEM map of native *Ax*qNOR at pH 8.0 determined by single-particle cryoEM to 2.6 Å resolution, showing the Coulomb potential of the scattered atoms. EM density for most transmembrane regions was well resolved for model building, except TM1 and TM7 which were visible only at very lower density thresholds. **(B)** Enlarged EM density of the binuclear centre highlighting heme b_3 and the side-chain orientations of His486, His537, His538, His629, Fe_B, Glu490, Glu494 and Glu559. **(C)** EM density revealing Cu atoms coordinated between periplasmic residues His217 and His224 when samples were vitrified on Quantifoil copper (Cu) grids. **(D)** Configuration and EM density of His217 and His224 when grids were prepared on Quantifoil gold (Au), where His224 adopts an alternative conformation and no Cu density is observed. *Ax*qNOR is depicted schematically as segmented rectangular box, with residues colour-coded according to the protein scaffold and the associated heme b_3.

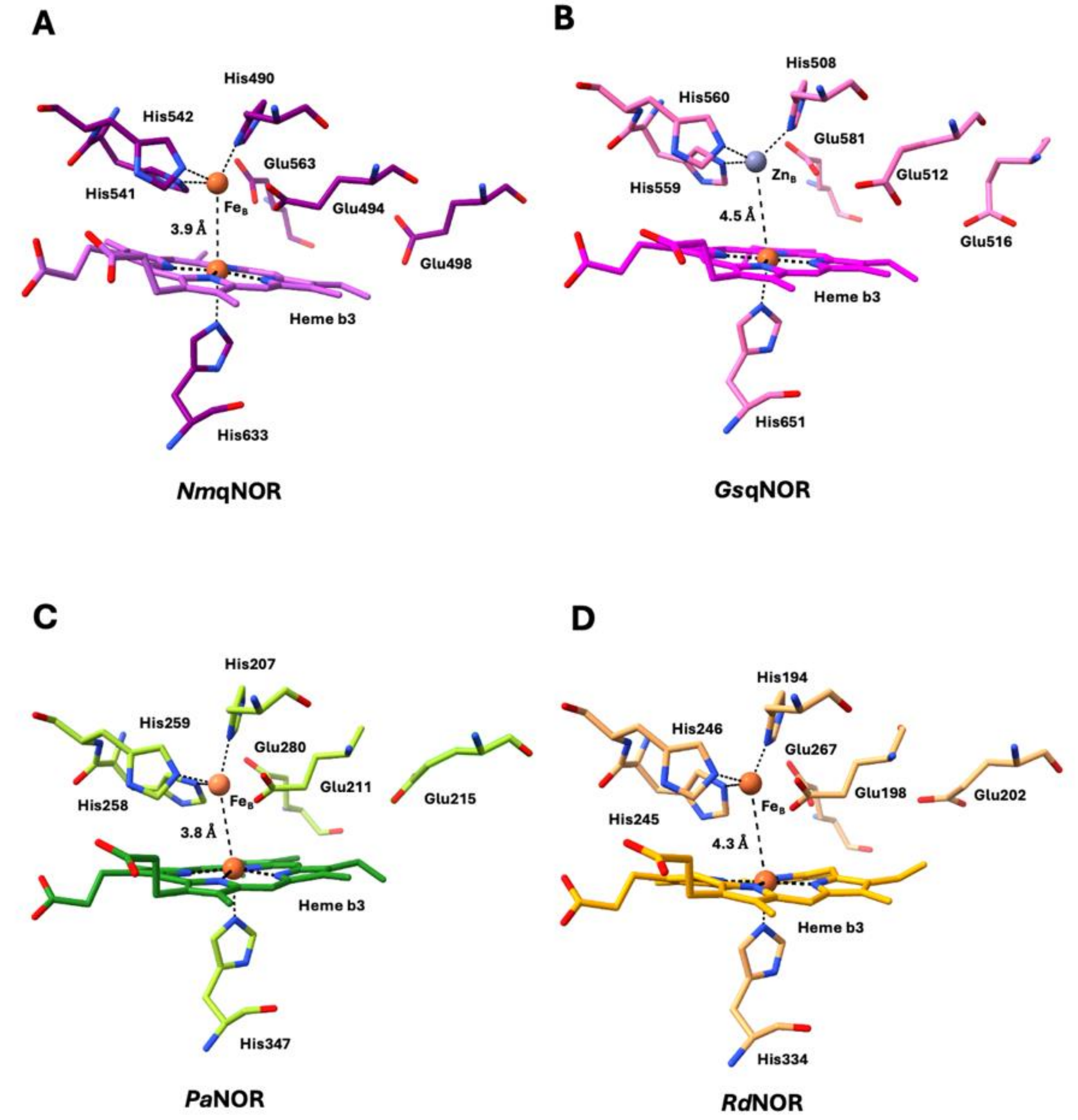

Supplementary Figure 2 | Active-site configurations in homologous Nitric oxide reductases (NORs). (A) Binuclear centre of *Neisseria meningitidis* qNOR (*Nm*qNOR; PDB 6L1X) with Fe_B coordinated by His490, His541 and His542 in a tetrahedral geometry. The distance between Fe_B and heme b_3 is 3.9 Å and the conserved catalytic Glu residues (Glu494, Glu498 and Glu563) are indicated. **(B)** Binuclear centre of *Geobacillus stearothermophilus* qNOR (*Gs*qNOR; PDB 3AYG) with Zn_B coordinated by His508, His559 and His560 in a tetrahedral geometry. The distance between Zn_B and heme b_3 is 4.5 Å and the conserved catalytic Glu residues (Glu512, Glu516 and Glu581) are indicated. **(C)** Binuclear centre of *Pseudomonas aeruginosa* cNOR (*Pa*cNOR; PDB 3O0R) with Fe_B coordinated by Glu211, His207, His258 and His259 in a slightly distorted trigonal-bipyramidal geometry. The distance between Fe_B and heme b_3 is 3.8 Å and the conserved catalytic Glu residues (Glu211, Glu215 and Glu280) are indicated. **(D)** Binuclear centre of *Roseobacter denitrificans* cNOR (*Rd*cNOR; PDB 4XYD) with Fe_B coordinated by His194, His245, His246 as well as with O1 and O2 of an unidentified ligand in an octahedral geometry. The distance between Fe_B and heme b_3 is 4.3 Å and the conserved catalytic Glu residues (Glu198, Glu202 and Glu267) are indicated.

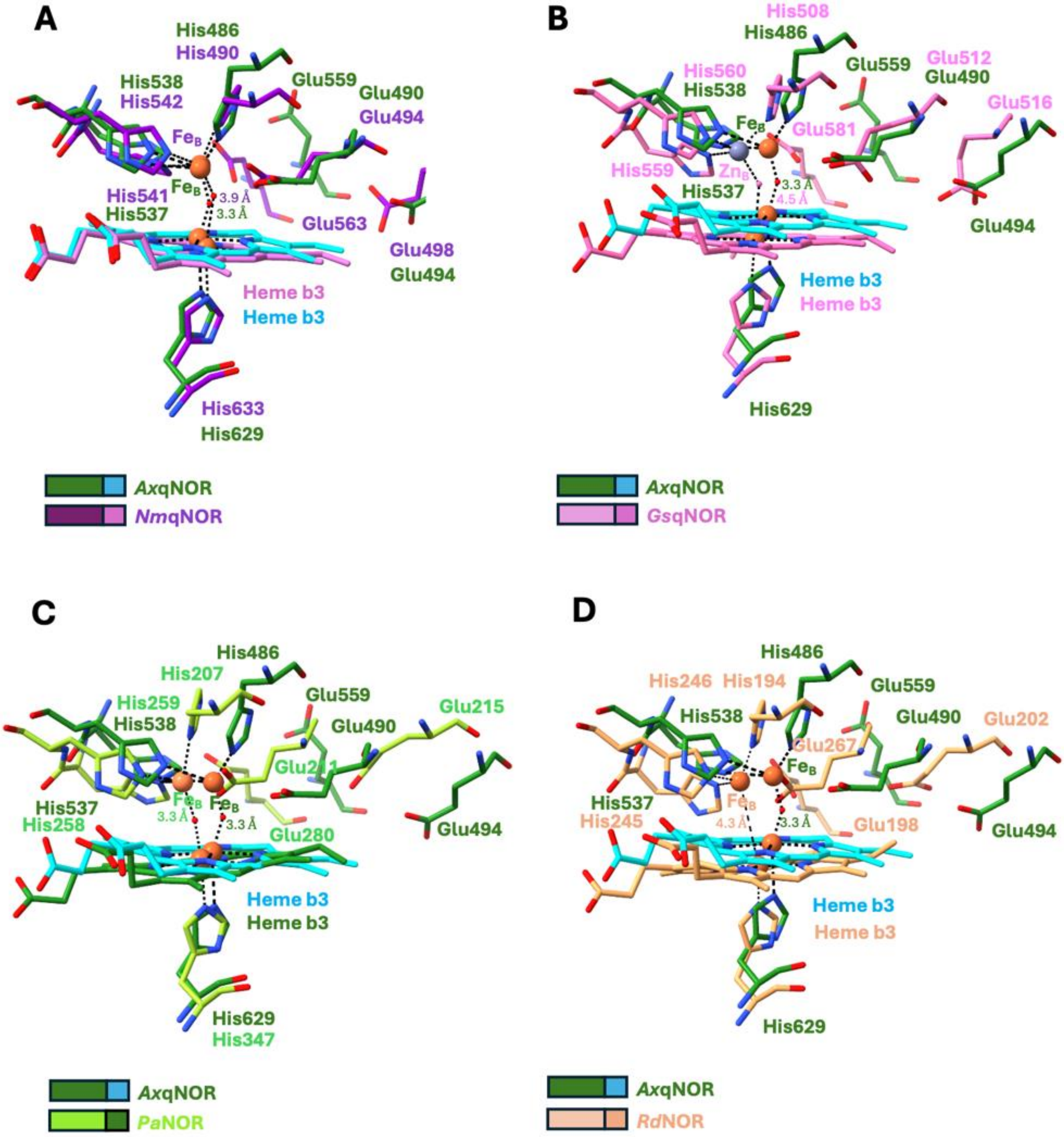

Supplementary Figure 3 | Active-sites superposition of native *Ax*qNOR and homologous Nitric oxide reductases (NORs). (A) *Ax*qNOR over *Neisseria meningitidis* qNOR (*Nm*qNOR; PDB 6L1X) **(B)** *Ax*qNOR over *Geobacillus stearothermophilus* qNOR (*Gs*qNOR; PDB 3AYG) **(C)** *Ax*qNOR over *Pseudomonas aeruginosa* cNOR (*Pa*cNOR; PDB 3O0R) **(D)** *Ax*qNOR over *Roseobacter denitrificans* cNOR (*Rd*cNOR; PDB 4XYD). NOR structures are depicted schematically as segmented rectangular boxes, with residues colour-coded according to the protein scaffold and the associated heme b_3.

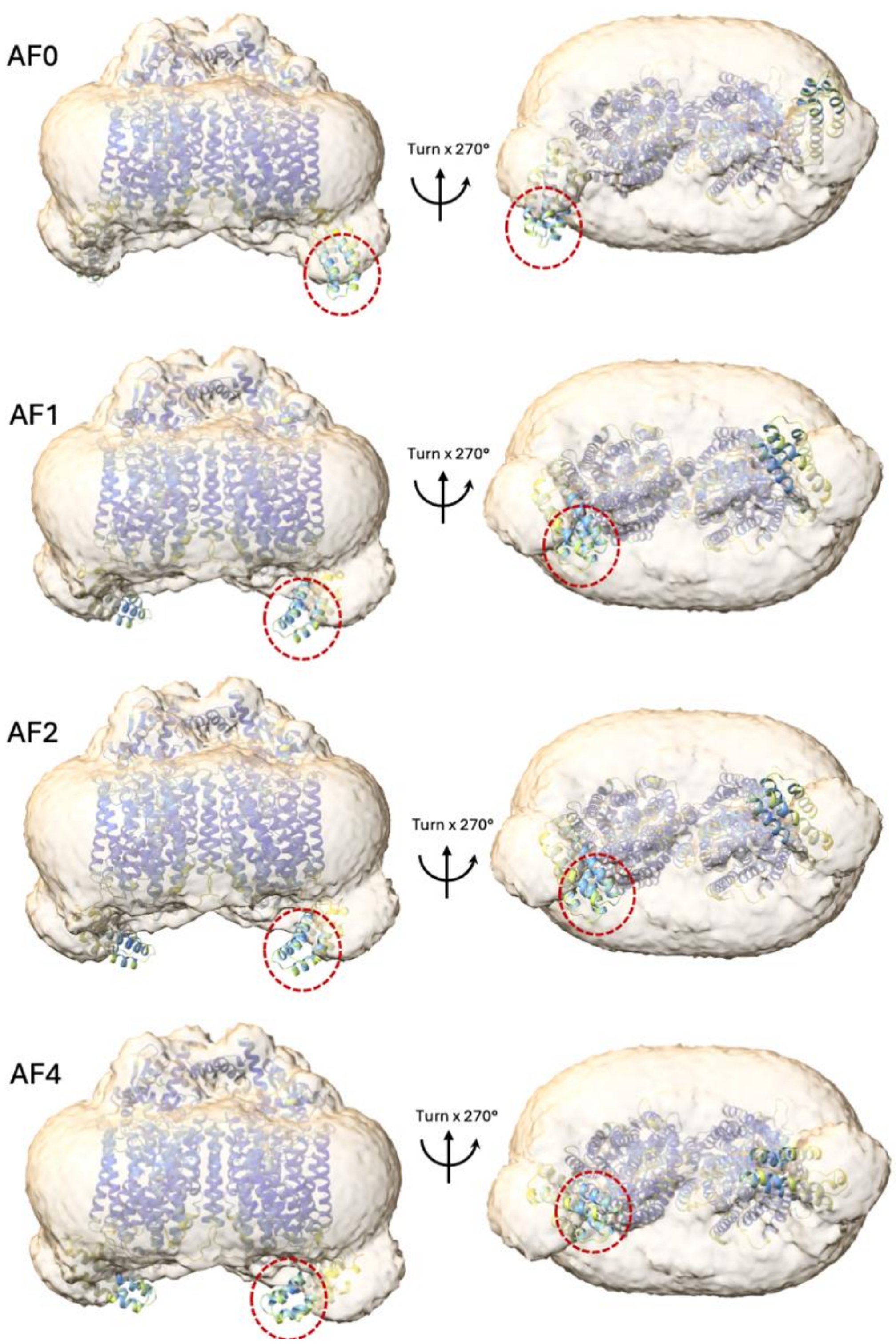

Figure S4 | Structural modelling of AlphaFold3 BRIL-*Ax*qNOR predicted models into the cryoEM density map. The BRIL-*Ax*qNOR cryoEM map is shown in the plane of the lipid bilayer (left) and from the cytoplasmic side (right), with rigid-body fitting of representative AlphaFold3 models-AF0, AF1, AF2, AF3, and AF4. Four out of five models exhibited poor fitting, with portions of the BRIL domain protruding outside the corresponding density is highlighted by red circle.

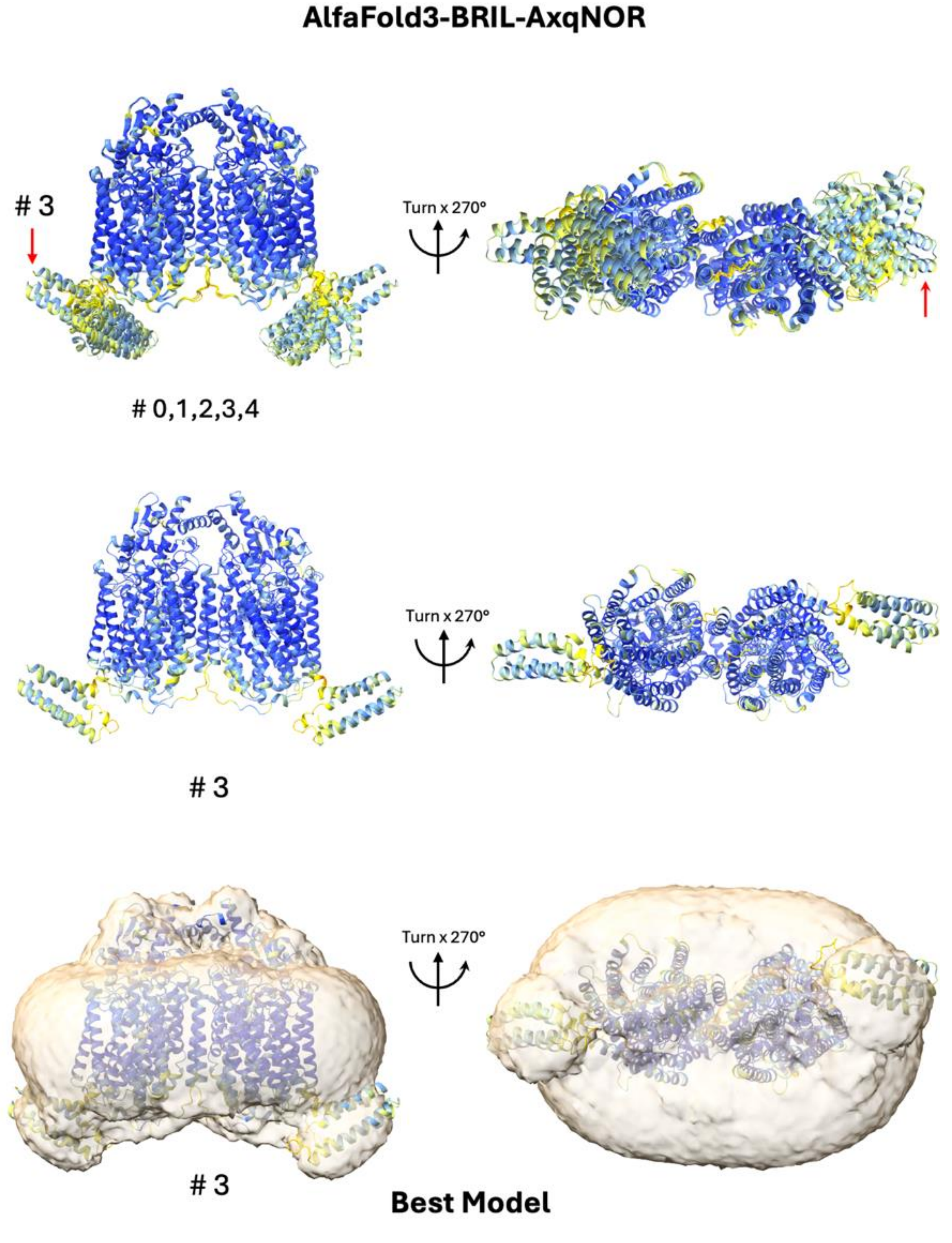

Figure S5 | Structural modeling of AlphaFold3 BRIL-*Axq*NOR predicted models into the cryoEM density map. AF3 displayed the highest model-to-map correlation coefficient (~0.7) upon rigid-body fitting (density contoured at 0.15 σ), providing the most accurate overall fit and enabling improved placement of the BRIL domain within the EM density compared with the other predictions. Red arrow shows orientation of BRIL in different AlphaFold models.

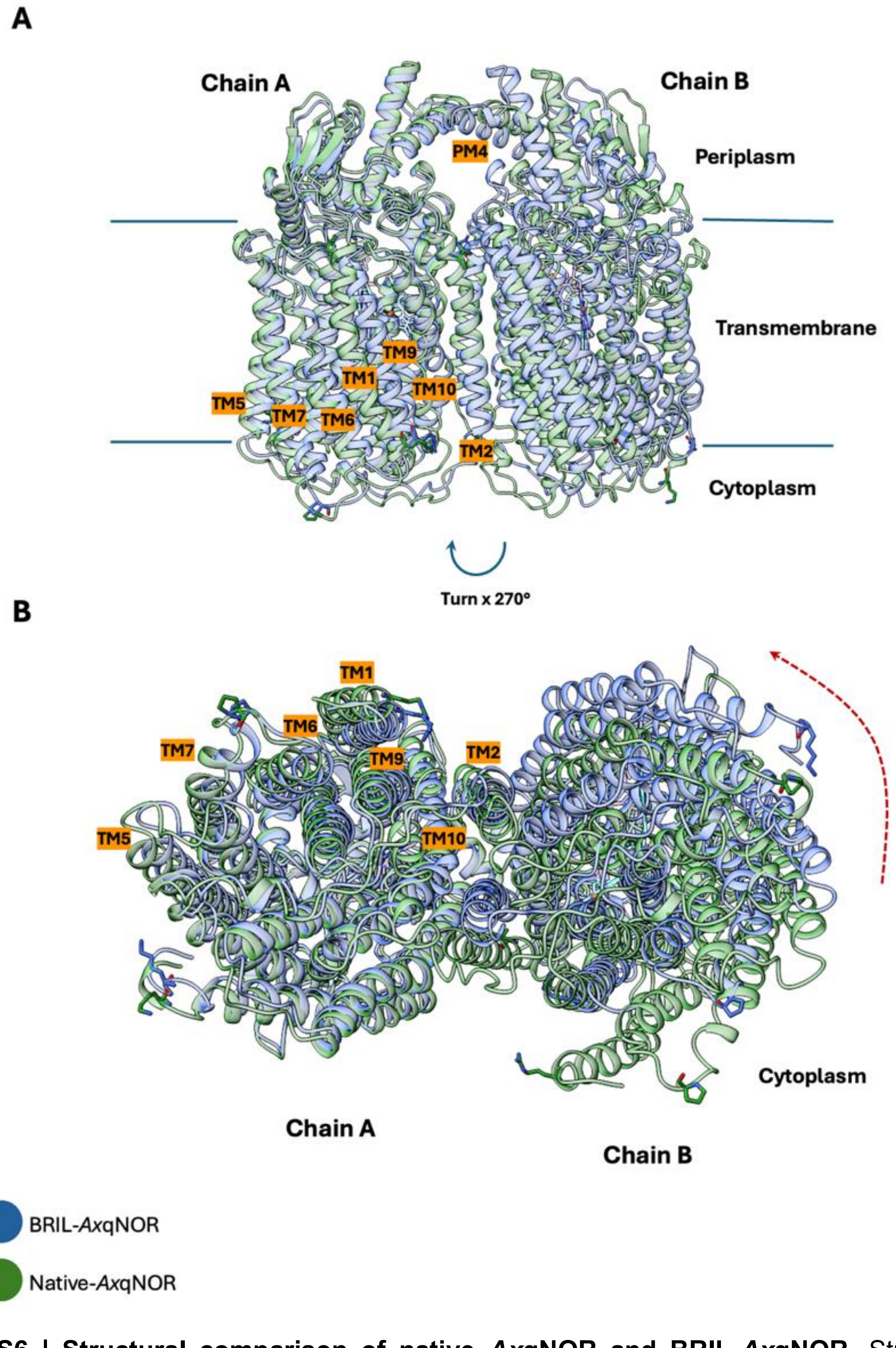

Figure S6 | Structural comparison of native *Ax*qNOR and BRIL-*Ax*qNOR. Structural superposition shows that the monomeric fold is largely preserved, with only subtle shifts in transmembrane helices TM1, TM2, TM5, TM6, TM7, TM9, and TM10, whereas the dimeric assembly undergoes pronounced conformational changes (movement indicated by red arrows). **(A)** Side view of *Ax*qNOR in the plane of the lipid bilayer. **(B)** View from the Periplasmic side. BRIL-*Ax*qNOR is shown in marine blue and native *Ax*qNOR in green at 50% transparency.

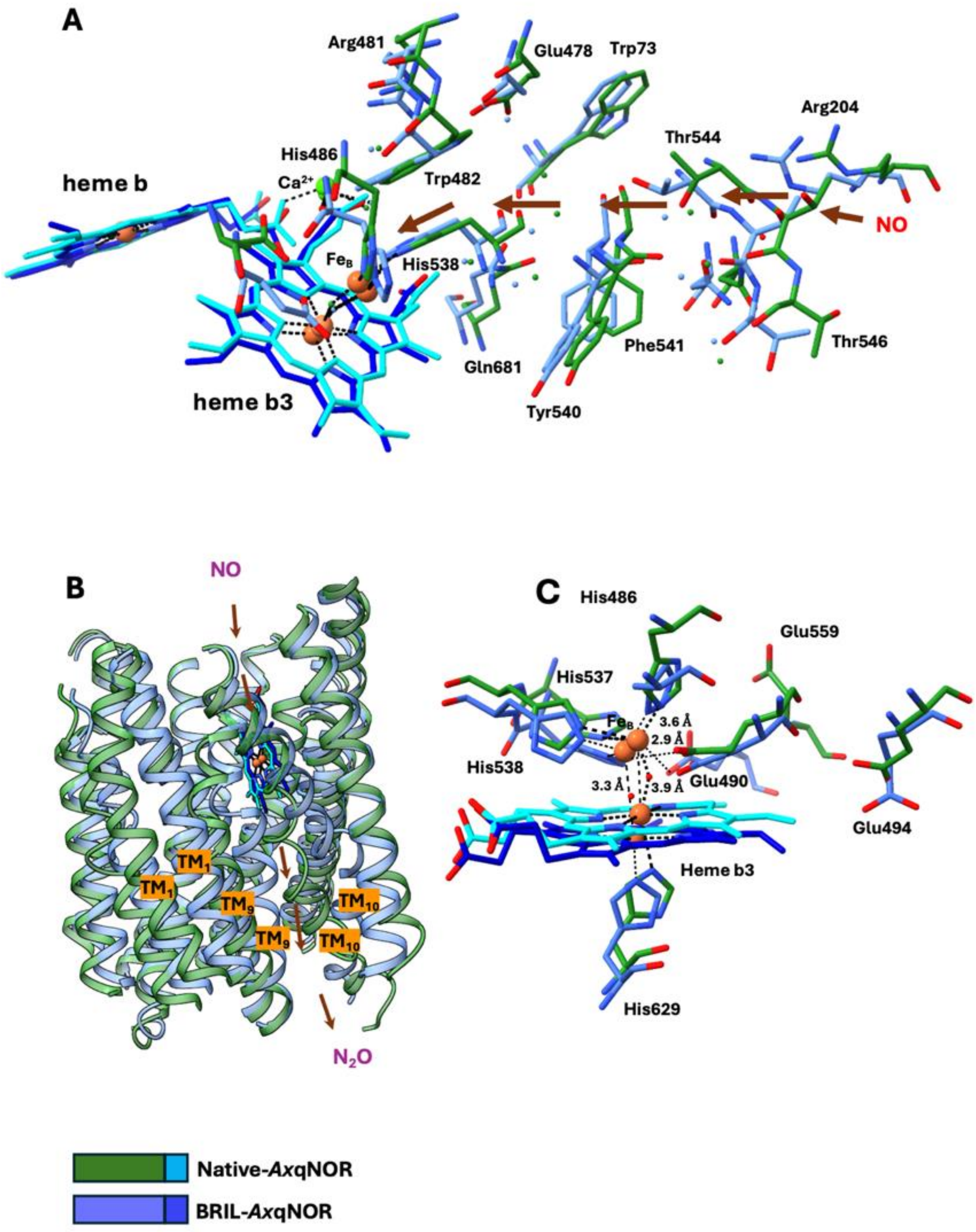

Figure S7 | Structural comparison of substrate entry, product release channels, and binuclear center in native and BRIL-*Ax*qNOR. Superposition of native *Ax*qNOR and BRIL-*Ax*qNOR reveals subtle differences in **(A)** the NO substrate entry channel, **(B)** the N_2O product release channel, and **(C)** the binuclear catalytic center, where the distance between Fe_B and heme b_3 increases from ~3.3 Å in native *Ax*qNOR to ~3.9 Å in BRIL-*Ax*qNOR. These minor structural variations may influence substrate entry, product release, or catalytic dynamics, potentially explaining the modest differences in NO reduction rates. BRIL-*Ax*qNOR is shown in marine blue and native *Ax*qNOR in green at 50% transparency. *Ax*qNOR is depicted schematically as segmented rectangular box, with residues colour-coded according to the protein scaffold and the associated heme b_3..

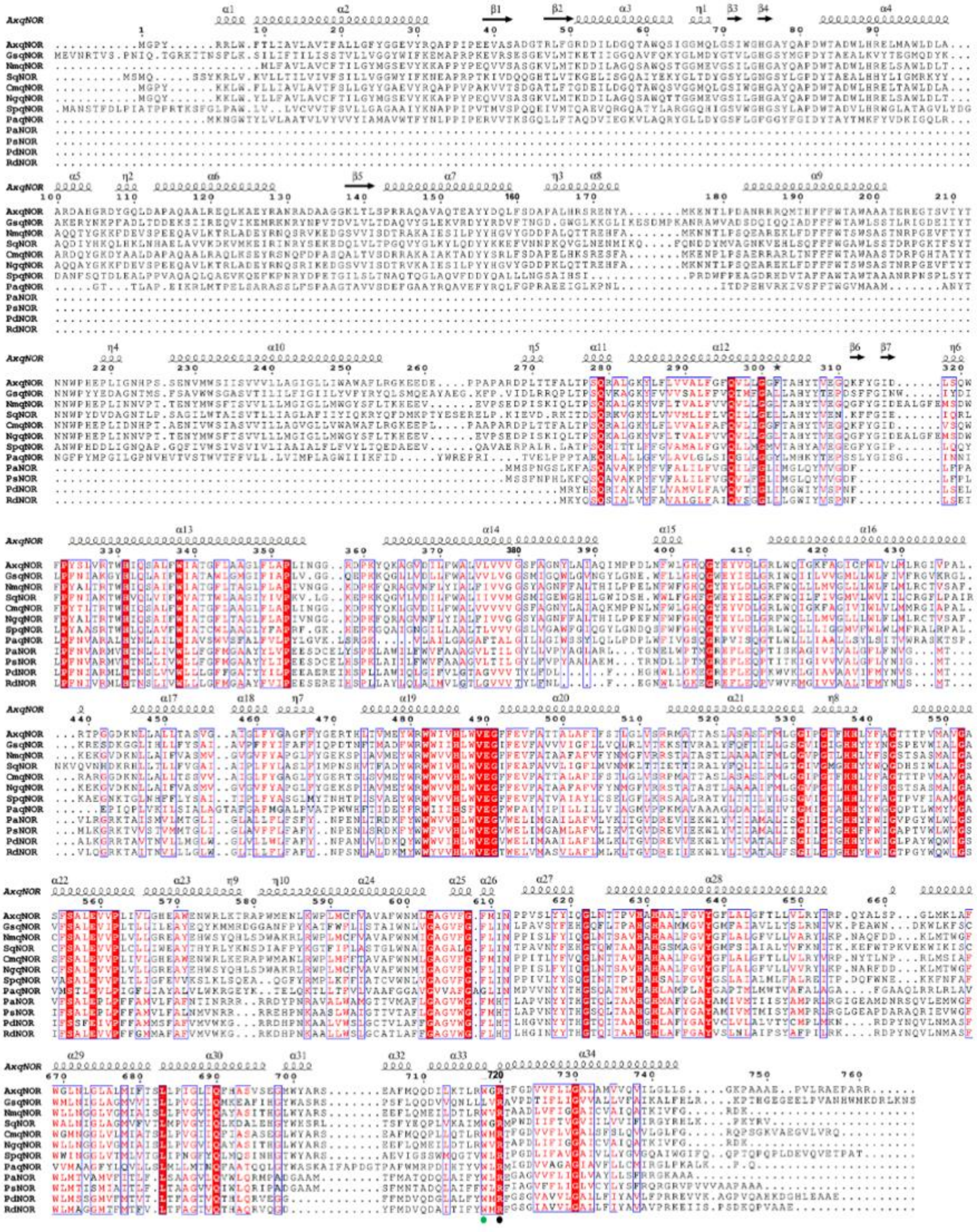

Figure S8: Multiple sequence alignment highlighting the conservation and variability of residues within NORs. Conserved and semi-conserved residues are indicated, including key positions Trp718 and Arg720 (marked by green and black circles, respectively). An exception is observed in *Geobacillus stearothermophilus*, where the conserved Trp718 (highlighted as green speher) is replaced by Leu. The qNOR sequences shown are from: (1) *Achromobacter xylosoxidans*, (2) *Geobacillus stearothermophilus*, (3) *Neisseria meningitidis*, (4) *Staphylococcus aureus*, (5) *Cupriavidus metallidurans*, (6) *Neisseria gonorrhoeae*, (7) *Synechocystis* sp., and (8) *Pyrobaculum aerophilum*. The cNOR sequences are from: (9) *Pseudomonas aeruginosa*, (10) *Pseudomonas denitrificans*, (11) *Pseudomonas stutzeri*, and (12) *Roseobacter denitrificans*.

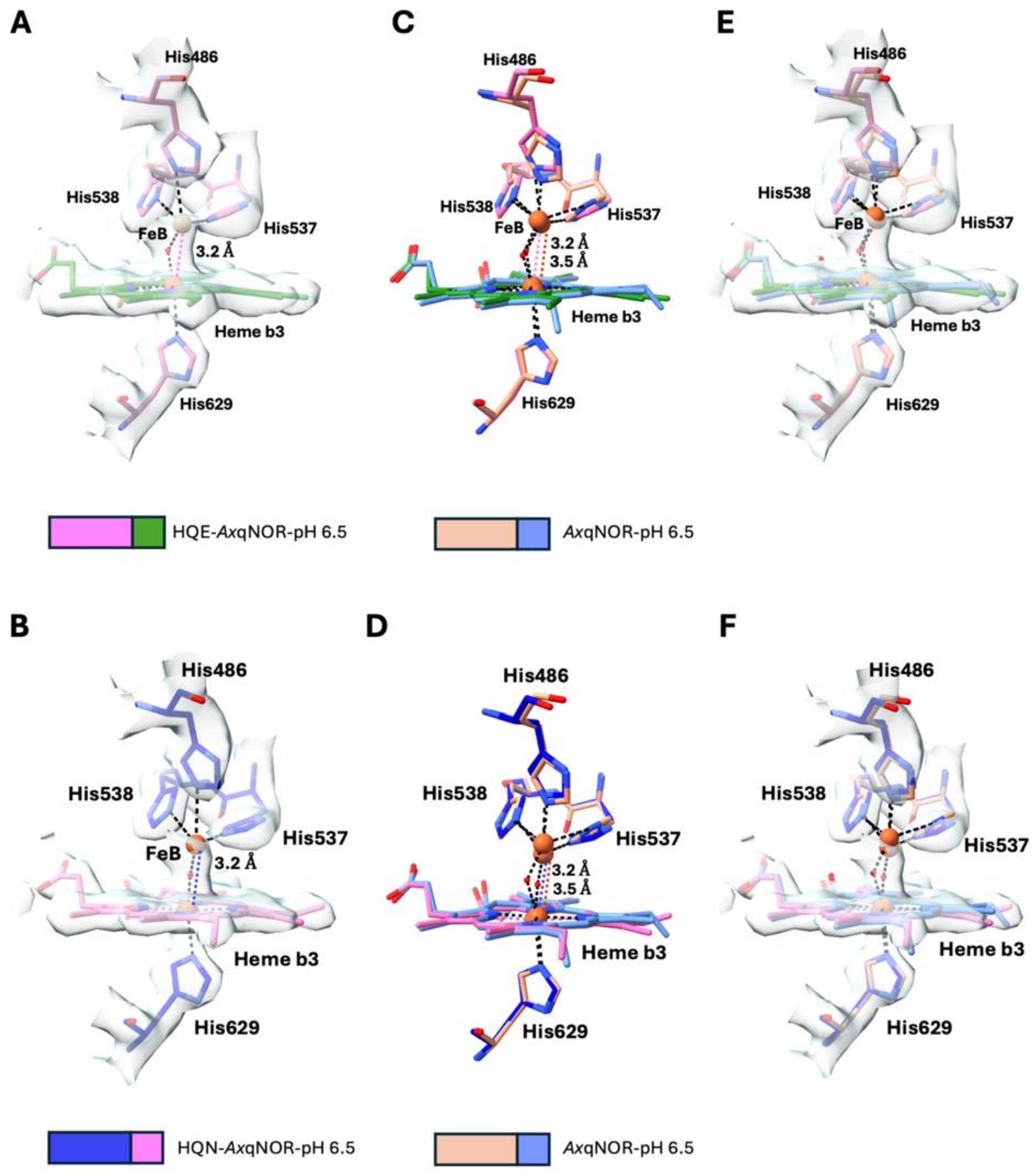

Figure S9. Structural comparison of the binuclear active site in quinol- and hydroxyquinol-bound *Ax*qNOR at pH 6.5. (A, B) cryoEM density maps of the binuclear active site in quinol-bound (HQE) and hydroxyquinol-bound (HQN) *Ax*qNOR, showing the coordination environment of heme b_3 and Fe_B. **(C, D)** Superpositions of apo *Ax*qNOR with HQE- or HQN-*Ax*qNOR at pH 6.5, highlighting the positions of heme b_3, Fe_B, and coordinating histidines (His486, His537, His538, His629), with Fe_B- heme b_3 distances indicated. **(E, F)** Corresponding cryoEM density maps of HQE- and HQN-*Ax*qNOR active sites, demonstrating preservation of the binuclear center upon ligand binding. *Ax*qNOR is depicted schematically as a segmented rectangular box, with residues color-coded according to the protein scaffold and the associated heme b_3 .

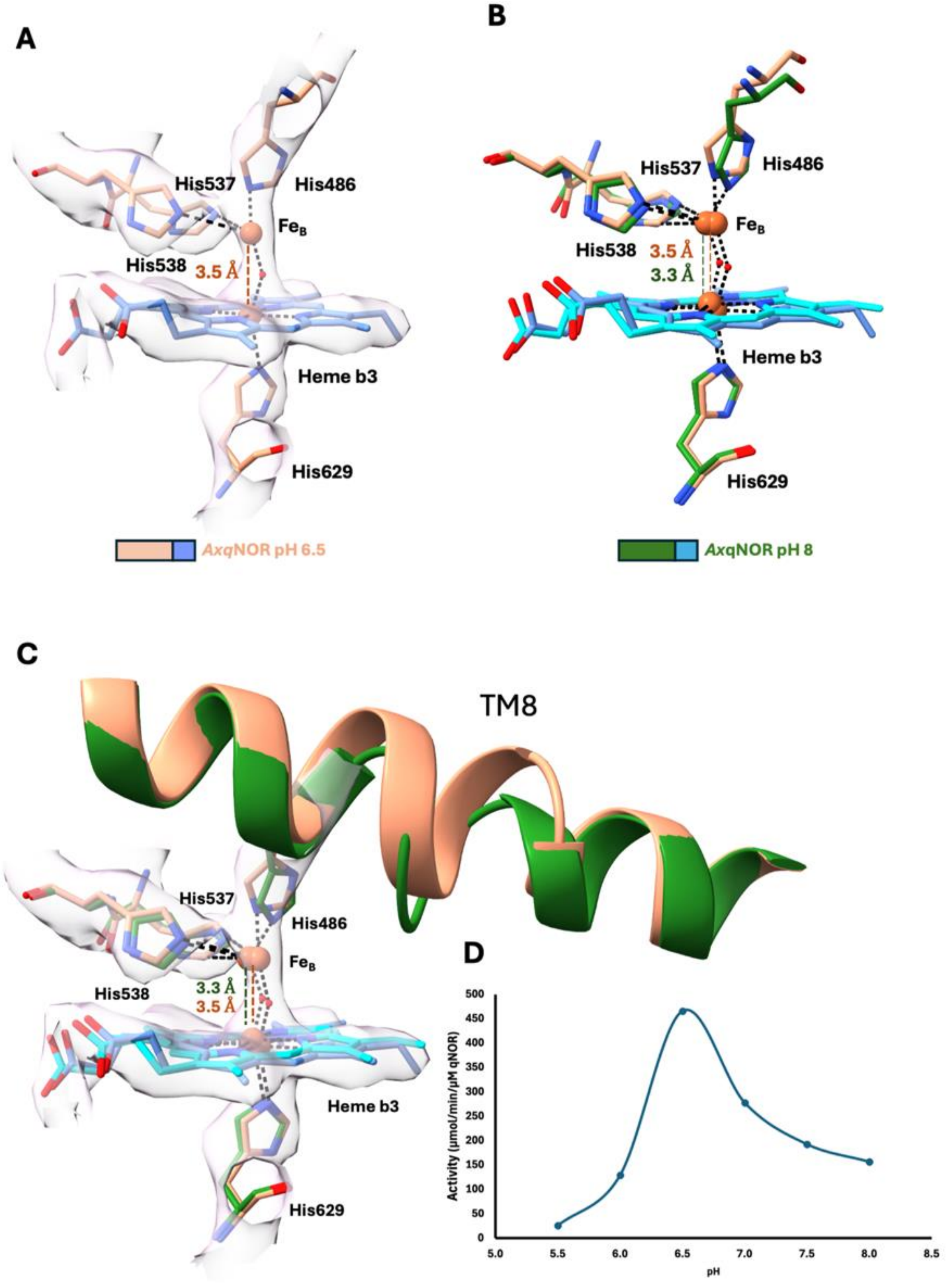

Figure S10 | Structure and features of native *Ax*qNOR at pH 6.5 (A) cryoEM density map of the binuclear active site in *Ax*qNOR at pH 6.5. **(B)** Superposition of native *Ax*qNOR at pH 6.5 and pH 8.0, highlighting the positions of heme b_3, Fe_B, and coordinating histidines (His486, His537, His538, His629), with Fe_B- heme b_3 distances indicated. **(C)** Corresponding cryoEM density map of the active site, demonstrating preservation of the binuclear center at both pH 6.5 and pH 8.0. **(D)** pH-dependent NO-reduction activity of native *Ax*qNOR shows a bell-shaped profile, with maximal activity at pH 6.5 and a decline at pH 8.0. Residues are color-coded according to the protein scaffold and the associated heme b_3 .

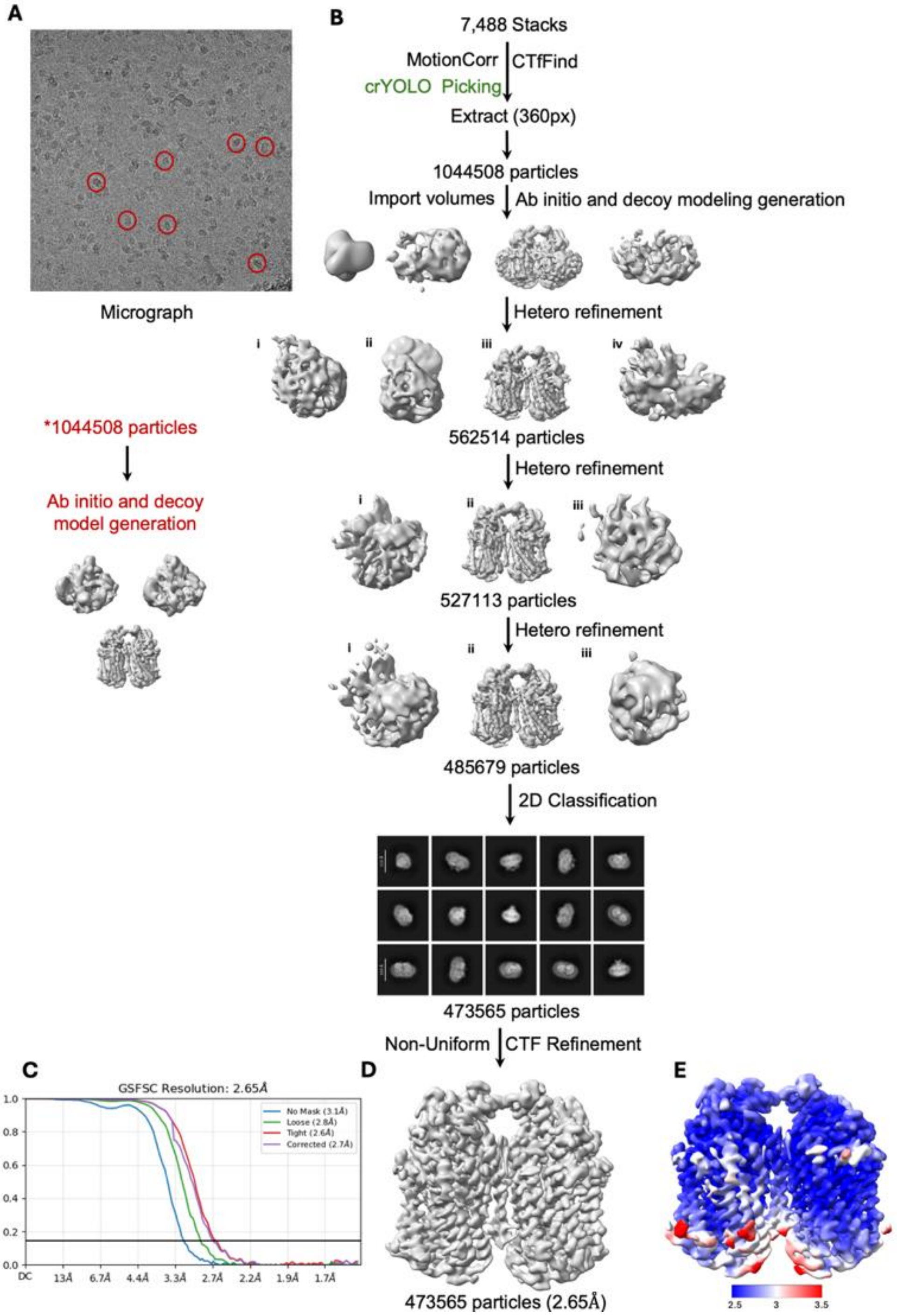

Figure S11: Processing workflow for the single-particle cryoEM analysis of native *Ax*qNOR at pH 8.0 on copper grid (A). Representative micrograph (left) showing native-*Ax*qNOR particles in red circles**.** (**B)** For the native *Ax*qNOR dataset at pH 8.0, 7,488 movies were motion-corrected in RELION, and particles were picked in crYOLO (~1 million particles) and imported into CryoSPARC for ab initio reconstruction and decoy model building. **(C, D)** Following multiple rounds of heterogeneous and 2D classification, ~473,000 particles were retained and refined using iterative global, local, and non-uniform refinement with C2 symmetry, yielding a 2.65 Å map. **(E)** Local resolution ranged from ~2.6 Å in transmembrane and periplasmic regions, including the active site, to ~4–5 Å in cytoplasmic-facing surfaces and connecting loops.

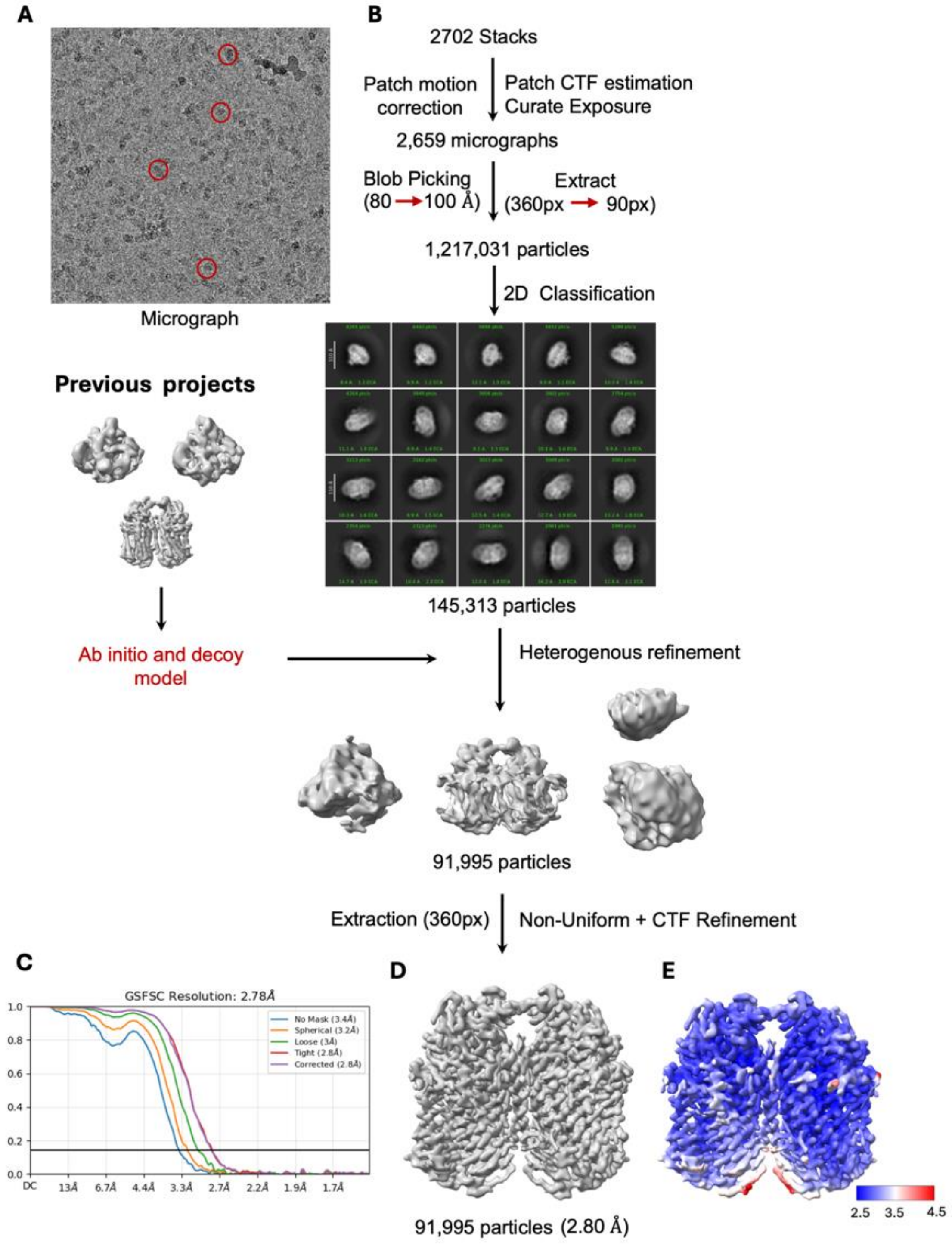

Figure S12: Single-Particle cryoEM Processing Workflow for *Ax*qNOR at pH 8 on a gold grid (A). Representative micrograph (left) showing *Ax*qNOR particles in red circles. **(B)** For the native-*Ax*qNOR dataset collected at pH 8 on Quantifoil Au aR1.2/1.3, 2702 movies were motion-corrected and CTF-estimated in CryoSPARC. Initial blob-based picking identified ~1.2 million particles, which after 2D classification and heterogeneous refinement were reduced to ~90,000 particles. These particles were eventually subjected to iterative global, local, and **(C, D)** non-uniform refinement with C2 symmetry, yielding a final map at 2.8 Å resolution. **(E)** Local resolution ranged from ~2.5 Å in transmembrane and periplasmic regions, including the active site, to ~3.0 Å in the cytoplasmic region.

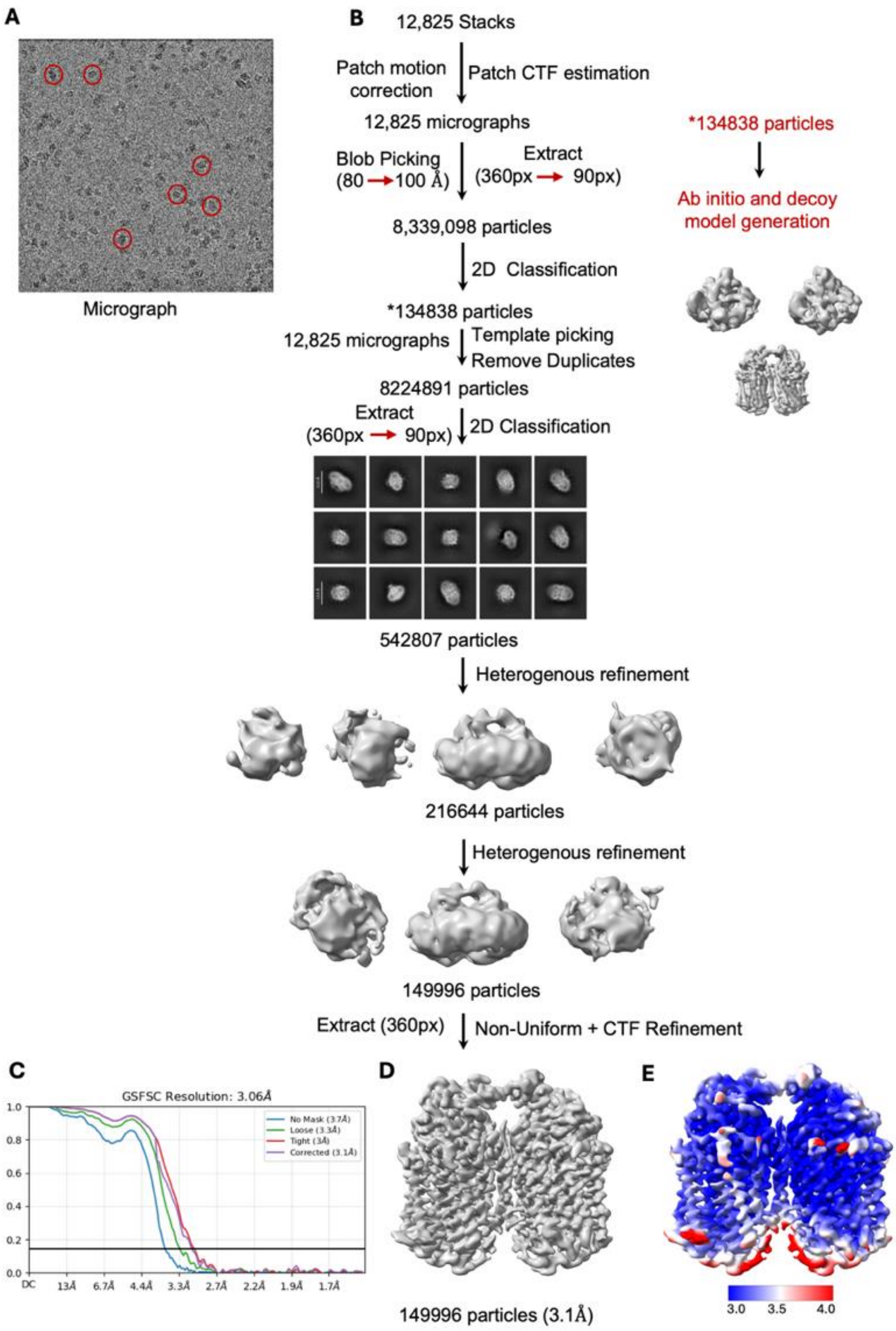

Figure S13: Processing workflow for the single-particle cryoEM analysis of native *Ax*qNOR at pH 6.5 (A). Representative micrograph (left) showing native- *Ax*qNOR particles in red circles. **(B)** For the native *Ax*qNOR dataset at pH 6.5, 12,825 movies were motion-corrected and CTF-estimated in CryoSPARC. Blob- and template-based particle picking followed by multiple rounds of 2D, ab initio model building and heterogenous refinement reduced ~8.3 million initial particles to ~150,000 particles. **(C,D)** These were refined through iterative global, local, and non-uniform refinement with C2 symmetry, yielding a 3.1 Å map. **(E)** Local resolution ranged from ~3.0 Å in transmembrane and periplasmic regions, including the active site, to ~4–5 Å in the cytoplasmic region.

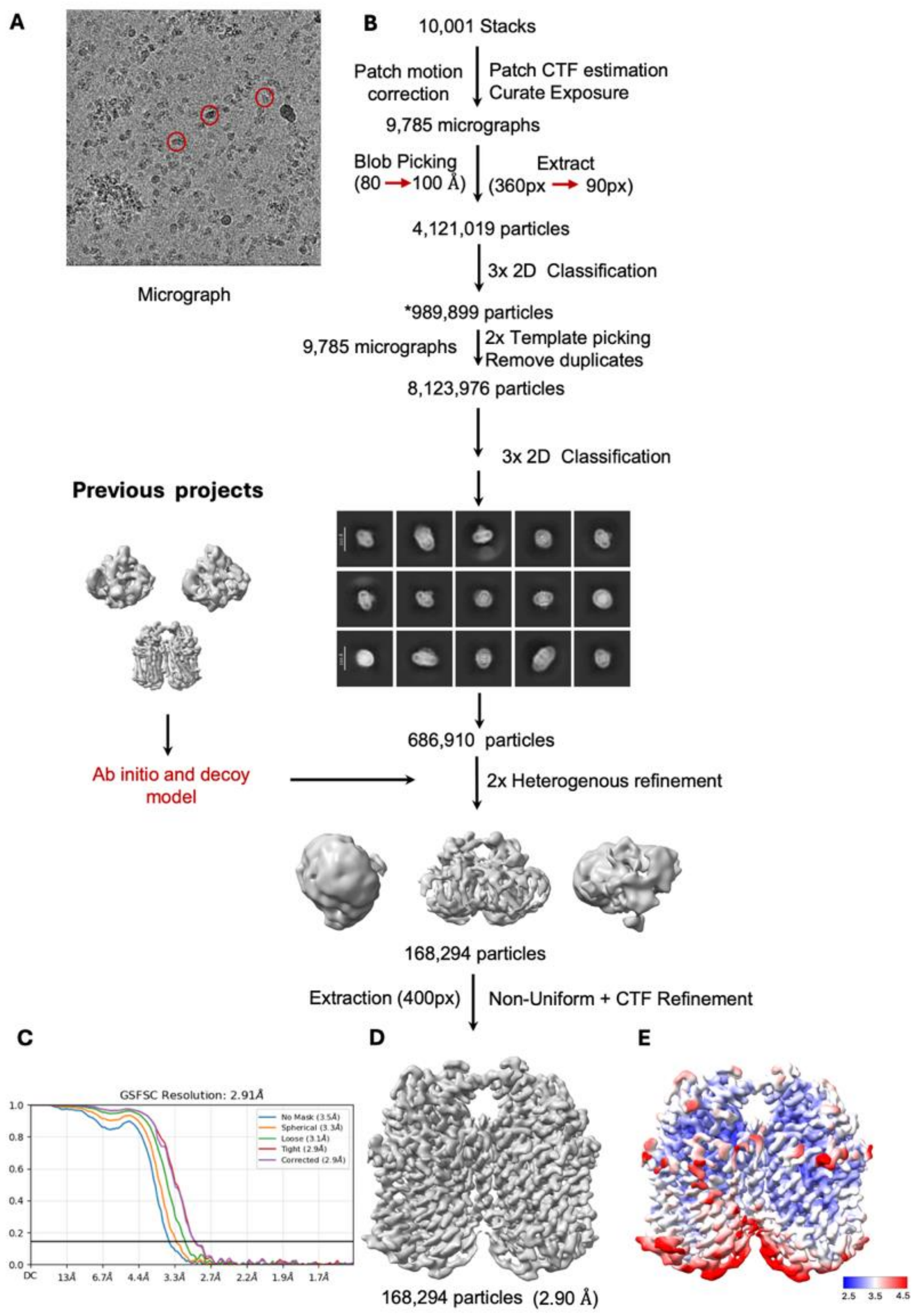

Figure S14: Single-Particle cryoEM Processing Workflow for *Ax*qNOR^{R720A} (A). Representative micrograph (left) showing *Ax*qNOR particles in red circles. **(B)** For the native-*Ax*qNOR^{R720A} dataset at pH 6.5, 10,001 movies were motion-corrected and CTF-estimated in CryoSPARC. Initial blob-based picking identified ~4 million particles, which after 2D classification were used for template-based picking. Finally, after removal of duplicate particles ~870,000 particles were retained for downstream processing. Extensive 2D classification and heterogeneous refined this to ~168,294 particles, which were subjected to iterative global, local, and **(C, D)** non-uniform refinement with C2 symmetry to produce a 2.9 Å map. **(E)** Local resolution ranged from ~2.7 Å in transmembrane and periplasmic regions, including the active site, to ~4.0 Å in the cytoplasmic region.

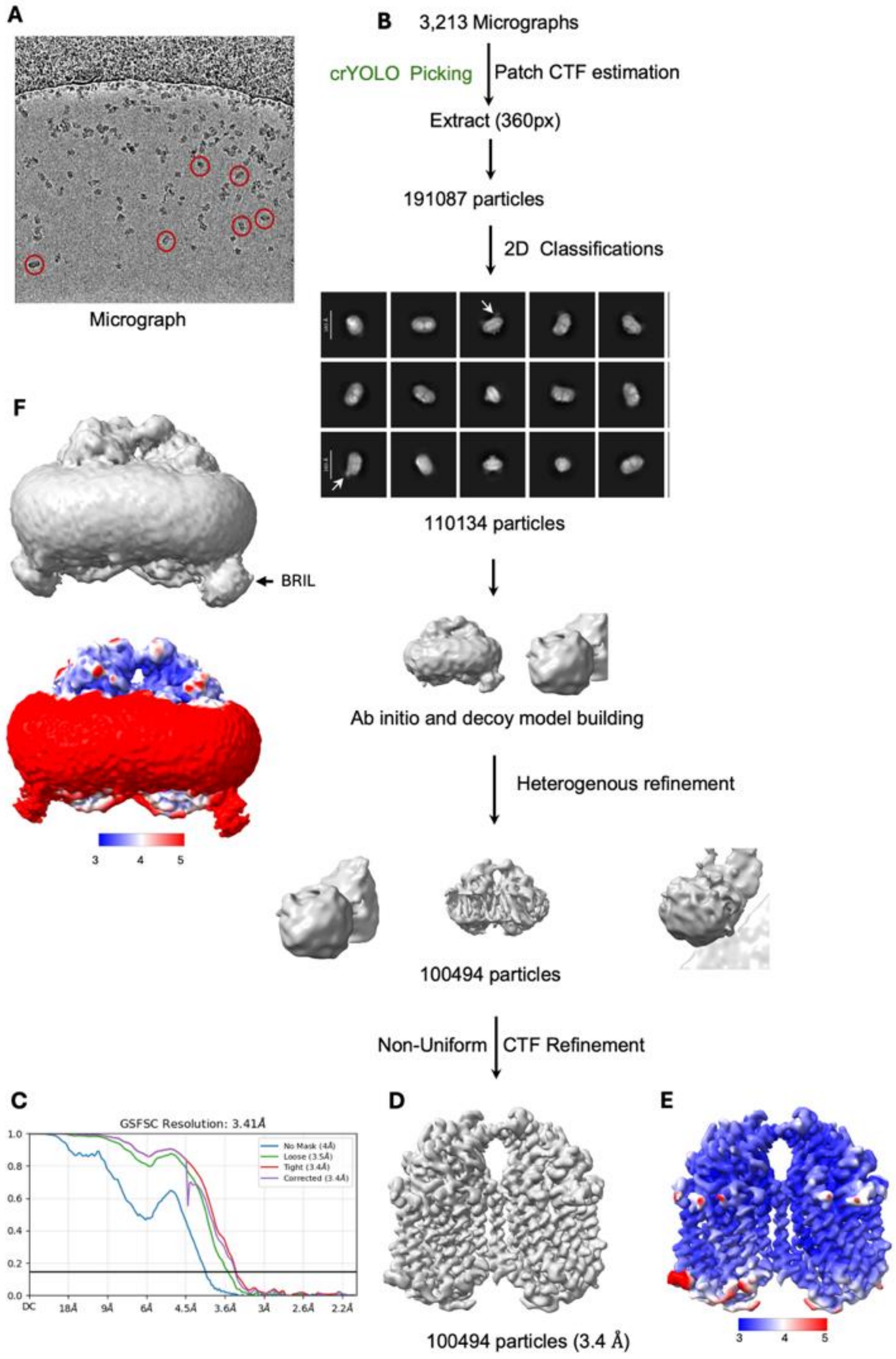

Figure S15: Processing workflow for the single-particle cryoEM analysis of BRIL-*Ax*qNOR (A). Representative micrograph (left) showing BRIL-*Ax*qNOR particles in red circles. **(B)** For the BRIL– *Ax*qNOR dataset, 3,213 motion-corrected micrographs were imported in CryoSPARC for PATCH ctf estimation and particle picking was performed in crYOLO, yielding ~190,000 particles. After 2D classification, ab initio reconstruction, and a single round of heterogeneous refinement, ~100,000 particles were retained. **(C, D)** These particles were refined through iterative global, local, and non-uniform refinement with C2 symmetry, producing a 3.4 Å map. **(E)** Local resolution ranged from ~3.4 Å in transmembrane and periplasmic regions, including the active site, to ~5–7 Å for the BRIL fusion. **(F)** 3D map showing local resolution of BRIL and its density at 0.25 σ in ChimeraX

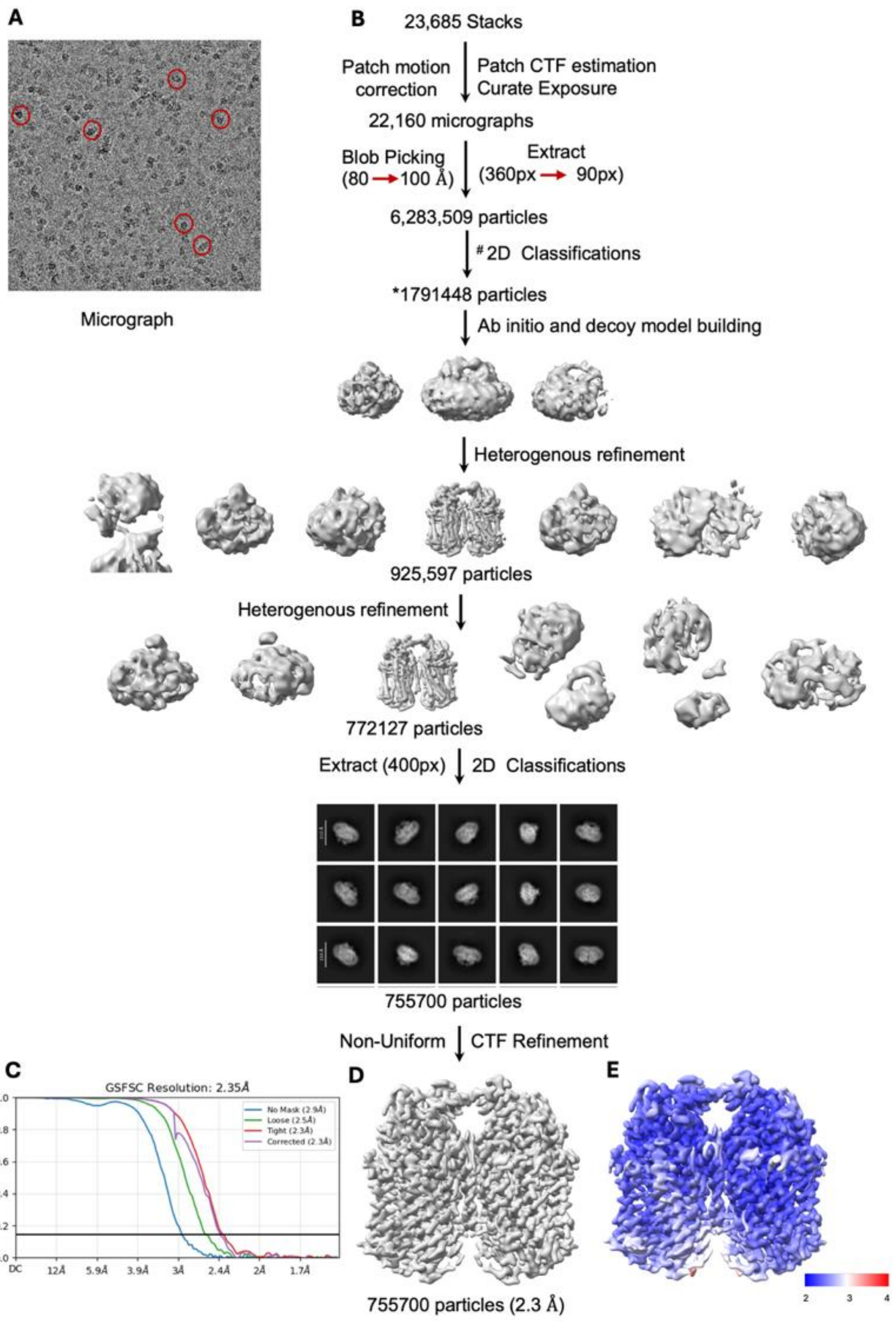

Figure S16: Processing workflow for the single-particle cryoEM analysis of native-*Ax*qNOR bound to hydroxyquinol (A). Representative micrograph (left) showing AxqNOR particles in red circles. **(B)** For the hydroxyquinol-bound native *Ax*qNOR dataset at pH 6.5 (*Ax*qNOR –HQN), 23,685 movies were motion-corrected and CTF-estimated in CryoSPARC. Initial blob-based picking identified ~6.3 million particles, which after multiple rounds of 2D classification, ab initio reconstruction and heterogenous refinement were reduced to ~770,000 particles. **(C, D)** These were refined through additional 2D classification and iterative global, local, and non-uniform refinement with C2 symmetry, yielding a 2.3 Å map. **(E)** Local resolution ranged from ~2.3 Å in transmembrane and periplasmic regions, including the active site, to ~3.0 Å in the cytoplasmic region.

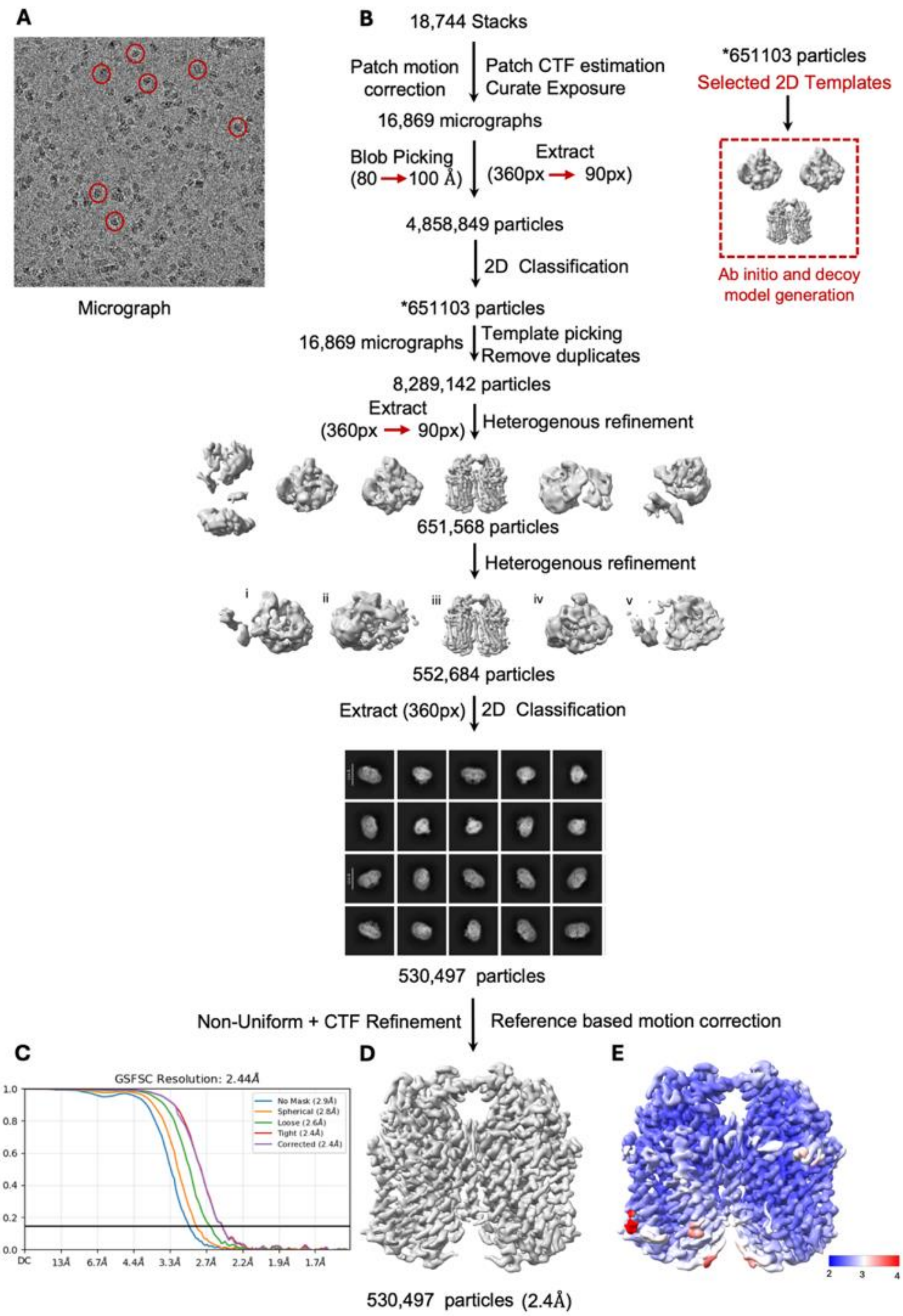

Figure S17: Processing workflow for the single-particle cryoEM analysis of native-*Ax*qNOR bound to quinol (A). Representative micrograph (left) showing *Ax*qNOR particles in red circles. **(B)** For the quinol-bound native *Ax*qNOR dataset at pH 6.5 (*Ax*qNOR-HQE), 18,744 movies were motion-corrected and CTF-estimated in CryoSPARC. Initial blob-based picking identified ~4.8 million particles, which after 2D classification were used for template-based picking, yielding ~8.2 million particles. **(C, D)** Extensive heterogeneous and 2D classification refined this to ~530,000 particles, which were subjected to iterative global, local, and non-uniform refinement with C2 symmetry to produce a 2.4 Å map. **(E)** Local resolution ranged from ~2.3 Å in transmembrane and periplasmic regions, including the active site, to ~3.0 Å in the cytoplasmic region.

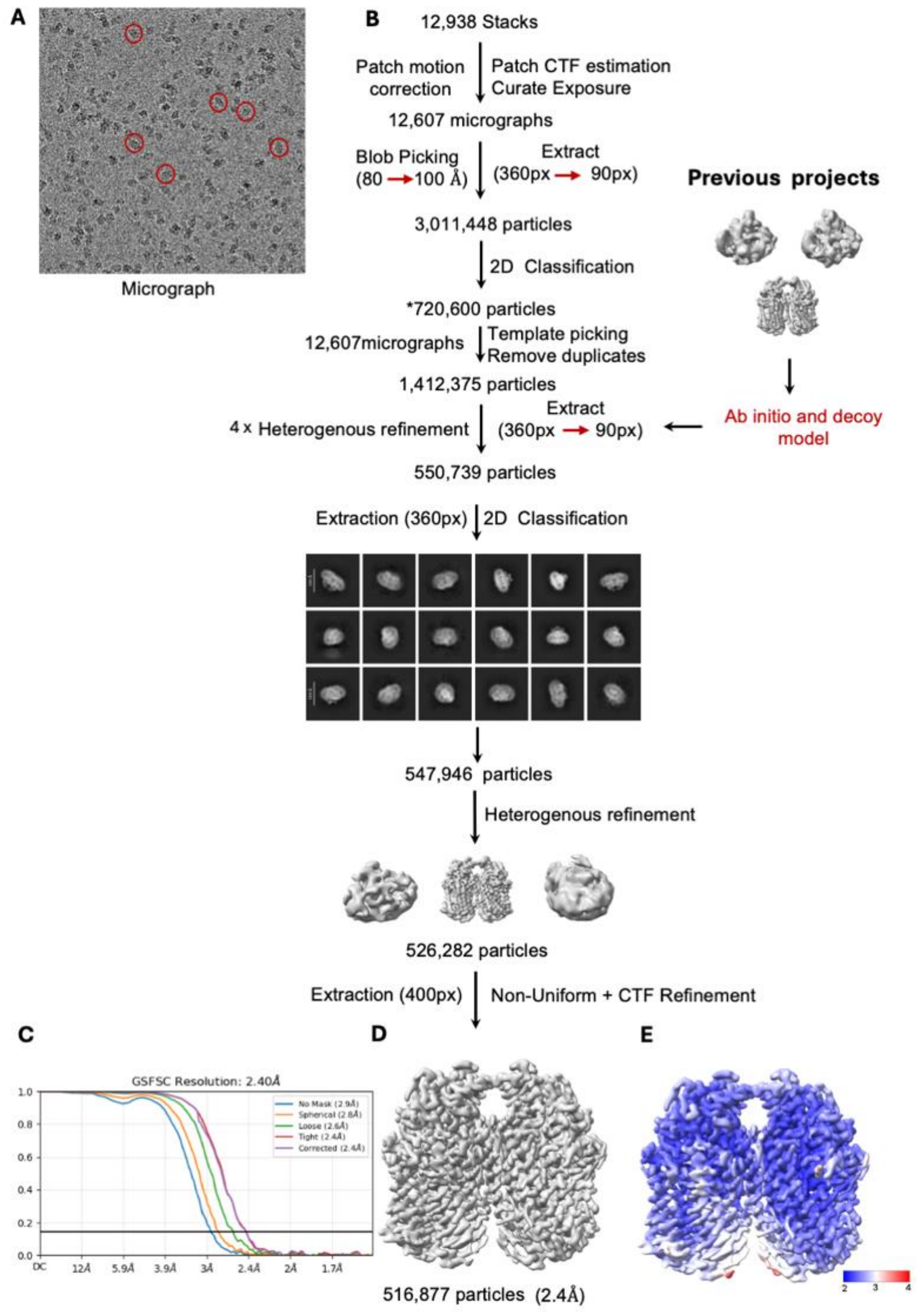

Figure S18: Single-Particle cryoEM Processing Workflow for *Ax*qNOR^{W718A} (A). Representative micrograph (left) showing *Ax*qNOR particles in red circles. **(B)** For the native-*Ax*qNOR^{W718A} dataset at pH 6.5, 12,938 movies were motion-corrected and CTF-estimated in CryoSPARC. Initial blob-based picking identified ~3 million particles, which after 2D classification were used for template-based picking. Finally, after removal of duplicate particles ~1.4 million particles were retained for downstream processing. Extensive 2D classification and heterogeneous refined this to ~500,000 particles, which were subjected to iterative global, local, and **(C, D)** non-uniform refinement with C2 symmetry to produce a 2.4 Å map. **(E)** Local resolution ranged from ~2.3 Å in transmembrane and periplasmic regions, including the active site, to ~3.0 Å in the cytoplasmic region.

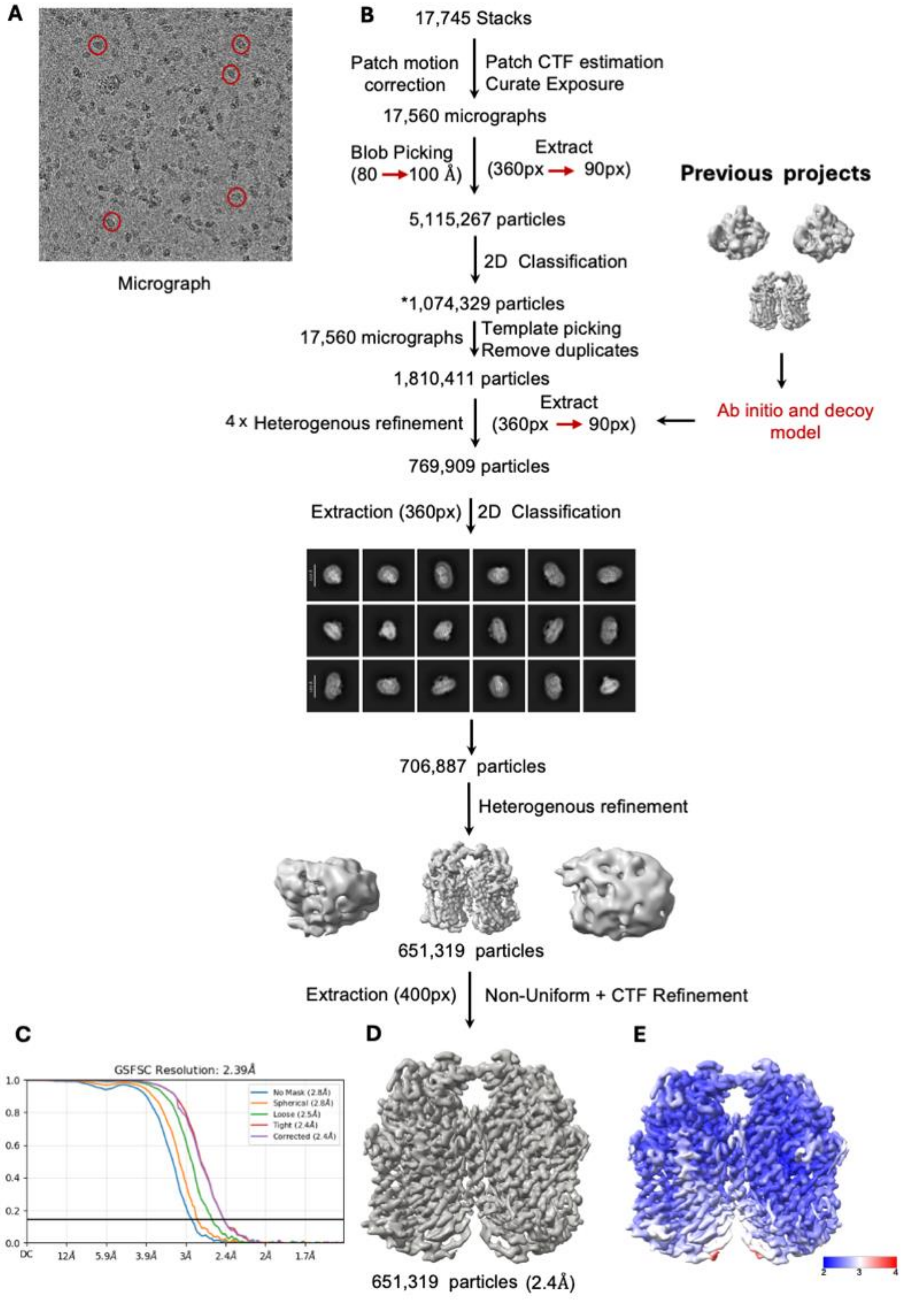

Figure S19: Single-Particle cryoEM Processing Workflow for *Ax*qNOR^{W718A} bound to quinol (A). Representative micrograph (left) showing AxqNOR particles in red circles. **(B)** For the quinol-bound native *Ax*qNOR^{W718A} dataset at pH 6.5 (*Ax*qNOR^{W718A}-HQE), 17,745 movies were motion-corrected and CTF-estimated in CryoSPARC. Initial blob-based picking identified ~5 million particles, which after 2D classification were used for template-based picking. Finally, after removal of duplicate particles ~1.8 million particles were retained for downstream processing. Extensive 2D classification and heterogeneous refined this to ~650,000 particles, which were subjected to iterative global, local, and **(C, D)** non-uniform refinement with C2 symmetry to produce a 2.4 Å map. **(E)** Local resolution ranged from ~2.3 Å in transmembrane and periplasmic regions, including the active site, to ~3.0 Å in the cytoplasmic region.

CryoEM data processing workflow and quality assessment

Data were processed using an integrated approach combining CryoSPARC (v4.6.0) and RELION (v4.0.2).[1,2]

For the native *Ax*qNOR dataset collected on Quantifoil Cu R1.2/1.3 at pH 8.0, a total of 7,488 movie stacks were subjected to motion correction using RELION's CPU-based implementation, followed by contrast transfer function (CTF) estimation with CTFFIND-4.1.[3] Particle picking was carried out in crYOLO using the pre-trained general model on the high-resolution Krios dataset, and the resulting coordinates were imported into RELION for particle extraction.[4,2] Approximately 1 million particles were initially extracted at a pixel size of 0.74 Å and transferred to CryoSPARC for *ab initio* model generation and decoy model building.[1] After several rounds of heterogeneous refinement, a subset of ~485,000 particles was selected and further curated by 2D classification, resulting in a final dataset of ~473,565 particles. These particles were subjected to multiple rounds of iterative refinement including global, local, and non-uniform refinement with C2 symmetry-yielding a final map at 2.65 Å resolution (Supplementary Figure S11 and Supplementary table 2). Local resolution estimation in CryoSPARC revealed that most of the transmembrane and periplasmic regions, including the active-site centre, were resolved to ~2.6 Å, whereas cytoplasmic-facing surfaces and connecting loops remained poorly resolved at ~4-5 Å.[1]

For the native *Ax*qNOR dataset collected on Quantifoil Au R1.2/1.3 at pH 8.0, a total of 2702 movie stacks were motion-corrected using Patch Motion Correction, followed by CTF estimation with Patch CTF in CryoSPARC.[1] Initial blob-based particle picking identified ~1.2 million particles, which were extracted with 4× binning. These particles were curated through multiple rounds of 2D classification, resulting in a subset of ~140,000 particles which were curated by one round of heterogenous refinement yielding ~ 91,995 particles. These were re-extracted at a box size of 400 px and refined through multiple rounds of iterative refinement-including global, local, and non-uniform refinement with C2 symmetry-yielding a 2.8 Å map (Supplementary Figure S12 and Supplementary table 2). Local resolution estimation in CryoSPARC indicated that most transmembrane and periplasmic regions, including the active-site centre, reached ~2.5 Å, while the cytoplasmic region was resolved to ~3.0 Å.[1]

For the native *Ax*qNOR dataset at pH 6.5, a total of 12,825 movie stacks were processed using Patch Motion Correction, followed by CTF estimation with Patch CTF in CryoSPARC.[1] Initial particle picking with a blob-based approach yielded ~8.3 million particles, which were extracted with 4× binning. These particles were subjected to 2D classification to generate templates that were subsequently used for template-based particle picking, *ab initio* reconstruction, and decoy model generation. Template-based picking identified ~8.2 million

particles, which were extensively curated through multiple rounds of 2D classification, resulting in a subset of ~540,000 particles. Multiple round of 3D curation by heterogeneous refinement was then performed to remove junk particles, yielding ~149,996 particles. These were re-extracted at a box size of 360 px and subjected to multiple rounds of iterative refinement-including global, local, and non-uniform refinement with C2 symmetry-producing a final map at 3.1 Å resolution (Supplementary Figure S13 and Supplementary table 3). Local resolution estimation in CryoSPARC indicated that most transmembrane and periplasmic regions, including the active-site centre, reached ~3.0 Å, whereas the cytoplasmic region remained poorly resolved at ~4-5 Å.[1]

For the native *Ax*qNOR Arg720Ala mutant dataset (*Ax*qNOR^{R720A}), a total of 10,001 movie stacks were motion-corrected using Patch Motion Correction, followed by CTF estimation with Patch CTF in CryoSPARC.[1] Initial blob-based particle picking identified ~4.1 million particles, which were extracted with 4× binning. These particles underwent 2D classification to generate templates that were subsequently used for template-based particle picking, Template-based picking yielded ~8.1 million particles, which were again extensively curated through multiple rounds of 2D classification, resulting in a subset of ~680,000 particles. Several rounds of 3D curation by heterogeneous refinement were performed at this stage, yielding ~168,294 particles These were re-extracted at a box size of 400 px and refined through multiple rounds of iterative refinement-including global, local, and non-uniform refinement with C2 symmetry-yielding a 2.9 Å map (Supplementary Figure S14 and Supplementary table 3). Local resolution estimation in CryoSPARC indicated that most transmembrane and periplasmic regions, including the active-site centre, reached ~2.7 Å, while the cytoplasmic region was resolved to ~3.5 Å.[1]

For the BRIL-*Ax*qNOR dataset, data processing was performed using 3,213 previously motion-corrected micrographs in RELION which were subsequently imported into CryoSPARC for Patch CTF estimation.[5,2,1] Particle picking was carried out in crYOLO, yielding ~190,000 particles that were extracted at a pixel size of 1.07 Å for 2D classification.[4] Distinct 2D class averages representing ~110,000 particles were selected for *ab initio* model generation and decoy model building. A single round of heterogeneous refinement was performed to remove junk particles during this 3D curation step, resulting in a final dataset of ~100,494 particles. The particles were extracted using a box size of 420px and subjected to multiple rounds of iterative refinement-including global, local, and non-uniform refinement with C2 symmetry-yielding a final map at 3.4 Å resolution (Supplementary Figure S15 and Supplementary table 4). Local resolution estimation in CryoSPARC indicated that most transmembrane and periplasmic regions, including the active-site centre, were resolved to ~3.4 Å, whereas the BRIL fusion remained poorly resolved at ~5-7 Å.[1]

For the hydroxyquinol-bound native *Ax*qNOR dataset (*Ax*qNOR-HQN), a total of 23,685 movie stacks were motion-corrected using Patch Motion Correction, followed by CTF estimation with Patch CTF in CryoSPARC.[1] Initial blob-based particle picking identified ~6.3 million particles, which were extracted with 4× binning. Multiple rounds of 2D classification reduced this to ~1.8 million clean particles, which were subsequently used for *ab initio* reconstruction and decoy model generation. Several rounds of 3D curation by heterogeneous refinement were performed, yielding ~770,000 particles. These were re-extracted at a box size of 400 px and subjected to an additional 2D classification step, resulting in ~755,700 particles. The final set of particles underwent multiple rounds of iterative refinement-including global, local, and non-uniform refinement with C2 symmetry-yielding a 2.3 Å map (Supplementary Figure S16 and Supplementary table 5). Local resolution estimation in CryoSPARC indicated that most transmembrane and periplasmic regions, including the active-site centre, reached ~2.3 Å, while the cytoplasmic region was resolved to ~3.0 Å.[1]

For the quinol-bound native *Ax*qNOR dataset (*Ax*qNOR-HQE), a total of 18,744 movie stacks were motion-corrected using Patch Motion Correction, followed by CTF estimation with Patch CTF in CryoSPARC.[1] Initial blob-based particle picking identified ~4.8 million particles, which were extracted with 4× binning. These particles underwent 2D classification to generate templates that were subsequently used for template-based particle picking, *ab initio* reconstruction, and decoy model generation. Template-based picking yielded ~5.2 million particles, which were extensively curated through multiple rounds of heterogeneous refinement, resulting in a subset of ~550,000 particles. These were re-extracted at a box size of 360 px and subjected to an additional 2D classification step, yielding ~530,497 particles. The final set was refined through multiple rounds of iterative refinement-including global, local, and non-uniform refinement with C2 symmetry-yielding a 2.3 Å map (Supplementary Figure S17 and Supplementary table 5). Local resolution estimation in CryoSPARC indicated that most transmembrane and periplasmic regions, including the active-site centre, reached ~2.3 Å, while the cytoplasmic region was resolved to ~3.0 Å.[1]

For the native *Ax*qNOR Trp718Ala mutant dataset (***Ax*qNOR^{W718A}**), a total of 12,938 movie stacks were motion-corrected using Patch Motion Correction, followed by CTF estimation with Patch CTF in CryoSPARC.[1] Initial blob-based particle picking identified ~3 million particles, which were extracted with 4× binning. These particles were subjected to 2D classification to generate templates for subsequent template-based particle picking, yielding ~1.4 million particles. Extensive particle curation through multiple rounds of 3D heterogeneous refinement resulted in ~550,000 particles. These were re-extracted with a box size of 360 px and further cleaned by an additional round of 2D classification and heterogeneous refinement, producing a final set of ~516,877 particles. The particles were then re-extracted with a box size of 400

px and refined through multiple iterative refinement steps-including global, local, and non-uniform refinement with C2 symmetry-resulting in a 2.4 Å resolution map (Supplementary Figure S18 and Supplementary Table 6). Local resolution estimation in CryoSPARC indicated that most transmembrane and periplasmic regions, including the active-site center, reached ~2.3 Å resolution, whereas the cytoplasmic region was resolved to ~3.0 Å.[1]

For the native *Ax*qNOR Trp718Ala mutant dataset with quinol (*Ax*qNOR^{W718A} + quinol), a total of 17,745 movie stacks were motion-corrected using Patch Motion Correction, followed by CTF estimation with Patch CTF in CryoSPARC.[1] Initial blob-based particle picking identified ~5 million particles, which were extracted with 4× binning. These particles were subjected to 2D classification to generate templates for subsequent template-based particle picking, yielding ~6 million particles. Extensive particle curation through multiple iterative rounds of 2D classification and 3D heterogeneous refinement resulted in ~726,624 particles. These were re-extracted with a box size of 360 px and further cleaned by an additional round of 2D classification and heterogeneous refinement producing a final set of ~651,319 particles. The particles were then re-extracted with a box size of 400 px and refined through multiple iterative refinement steps-including global, local, and non-uniform refinement with C2 symmetry-resulting in a 2.4 Å resolution map (Supplementary Figure S19 and Supplementary Table 6). Local resolution estimation in CryoSPARC indicated that most transmembrane and periplasmic regions, including the active-site center, reached ~2.4 Å resolution, whereas the cytoplasmic region was resolved to ~3.0 Å.[1]

References:

(1) Punjani, A.; Rubinstein, J. L.; Fleet, D. J.; Brubaker, M. A. cryoSPARC: Algorithms for Rapid Unsupervised Cryo-EM Structure Determination. *Nat. Methods* **2017**, *14* (3), 290–296. https://doi.org/10.1038/nmeth.4169.
(2) Scheres, S. H. W. RELION: Implementation of a Bayesian Approach to Cryo-EM Structure Determination. *J. Struct. Biol.* **2012**, *180* (3), 519–530. https://doi.org/10.1016/j.jsb.2012.09.006.
(3) Rohou, A.; Grigorieff, N. CTFFIND4: Fast and Accurate Defocus Estimation from Electron Micrographs. *J. Struct. Biol.* **2015**, *192* (2), 216–221. https://doi.org/10.1016/j.jsb.2015.08.008.
(4) Wagner, T.; Merino, F.; Stabrin, M.; Moriya, T.; Antoni, C.; Apelbaum, A.; Hagel, P.; Sitsel, O.; Raisch, T.; Prumbaum, D.; Quentin, D.; Roderer, D.; Tacke, S.; Siebolds, B.; Schubert, E.; Shaikh, T. R.; Lill, P.; Gatsogiannis, C.; Raunser, S. SPHIRE-crYOLO Is a Fast and Accurate Fully Automated Particle Picker for Cryo-EM. *Commun. Biol.* **2019**, *2* (1), 218. https://doi.org/10.1038/s42003-019-0437-z.
(5) Gopalasingam, C. C.; Johnson, R. M.; Chiduza, G. N.; Tosha, T.; Yamamoto, M.; Shiro, Y.; Antonyuk, S. V.; Muench, S. P.; Hasnain, S. S. Dimeric Structures of Quinol-Dependent Nitric Oxide Reductases (qNORs) Revealed by Cryo-Electron Microscopy. *Sci. Adv.* **2019**, *5* (8), eaax1803. https://doi.org/10.1126/sciadv.aax1803.
